# Supplementary material for: Initiation of asexual reproduction by the AP2/ERF gene GEMMIFER in Marchantia polymorpha
Source: Curr Biol. 2026 May 4;36(9):2413–2423.e4. doi: 10.1016/j.cub.2026.03.083 (PMC13386412; doi:10.1016/j.cub.2026.03.083)
Supplement: Document S1. Figures S1–S6 and Table S1 [file mmc1.pdf]

Current Biology, Volume *36*

Supplemental Information

Initiation of asexual reproduction by the AP2/ERF gene *GEMMIFER* in *Marchantia polymorpha*

Go Takahashi, Saori Yamaya, Facundo Romani, Ignacy Bonter, Kimitsune Ishizaki, Masaki Shimamura, Tomohiro Kiyosue, Jim Haseloff, and Yuki Hirakawa

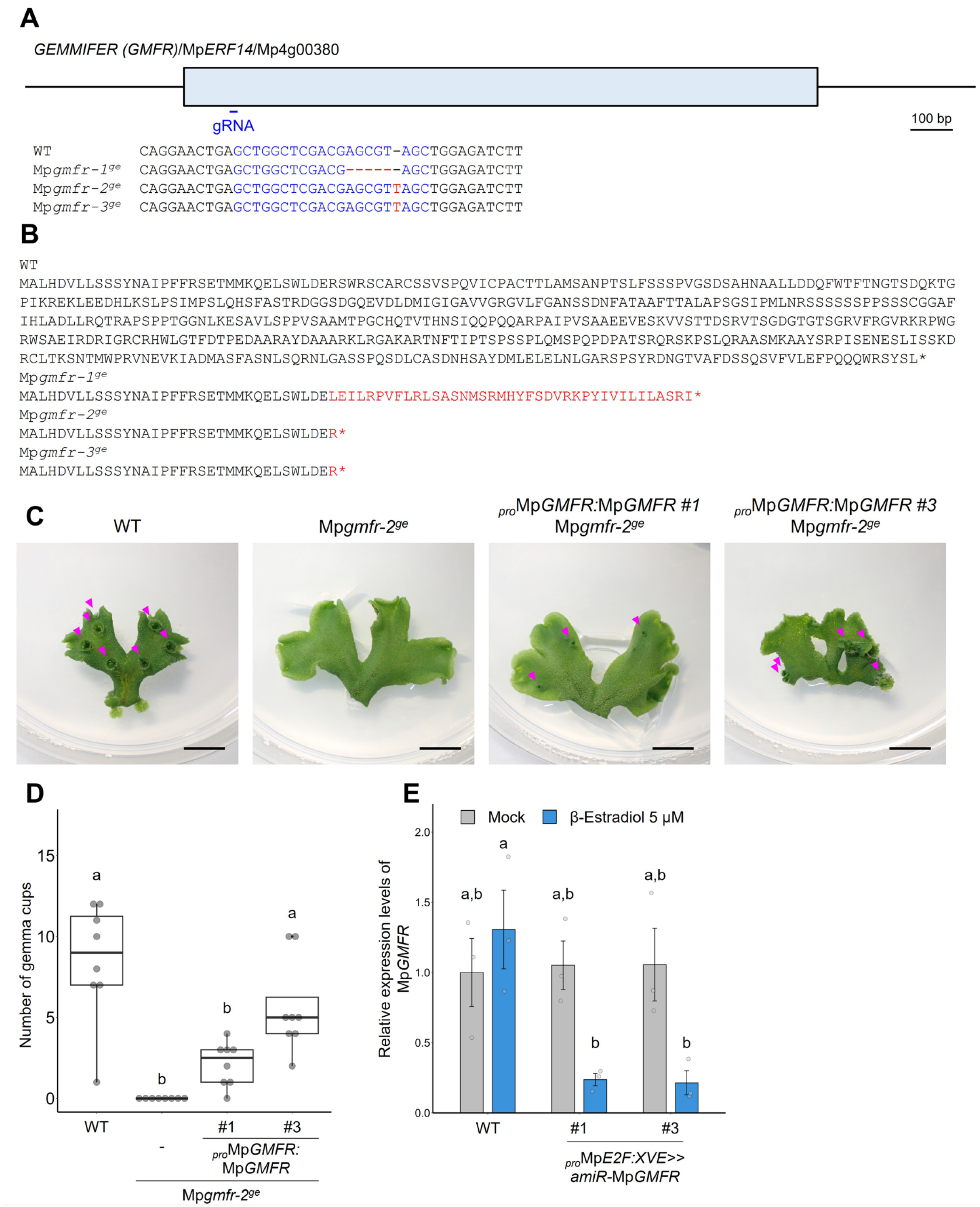

Figure S1. Loss-of-function mutants for *GEMMIFER*. Related to Figures 1 and 6.
(**A**) Structure of Mp*GMFR*/Mp*ERF14*/Mp4g00380 locus with the position of designed guide RNA (gRNA). An exon is shown as a box. Genotyping of genome editing alleles is indicated below. gRNA sequences are in blue. Deleted or inserted bases are in red. (**B**) Deduced MpGMFR protein sequences for WT and mutants. Sequences different from WT are in red. Asterisks indicate translational termination. (**C**) Complementation test for Mp*gmfr-2^{ge}* by gRNA-resistant Mp*GMFR* coding sequence driven under own promoter. Arrowheads indicate gemma cups. Scale bars, 1 cm. (**D**) The number of gemma cups (n=8). (**E**) Relative expression levels of Mp*GMFR* in 14-day-old plants of wild type and $_{pro}$Mp*E2F:XVE>>amiR*-Mp*GMFR* grown from gemmae in mock or β-estradiol-containing medium, normalized by Mp*APT* (n=3, biological replicates). In (**D**), the boxes show the median and interquartile range (IQR), and the whiskers extend to 1.5× IQR. Individual data points are plotted as dots. In (**E**), data are represented by mean and SD (bars) with individual data points (dots). Statistical significance was determined by two-way ANOVA with Tukey's post hoc test in (**D, E**). Means sharing the same superscript letters are not significantly different, $p < 0.05$.

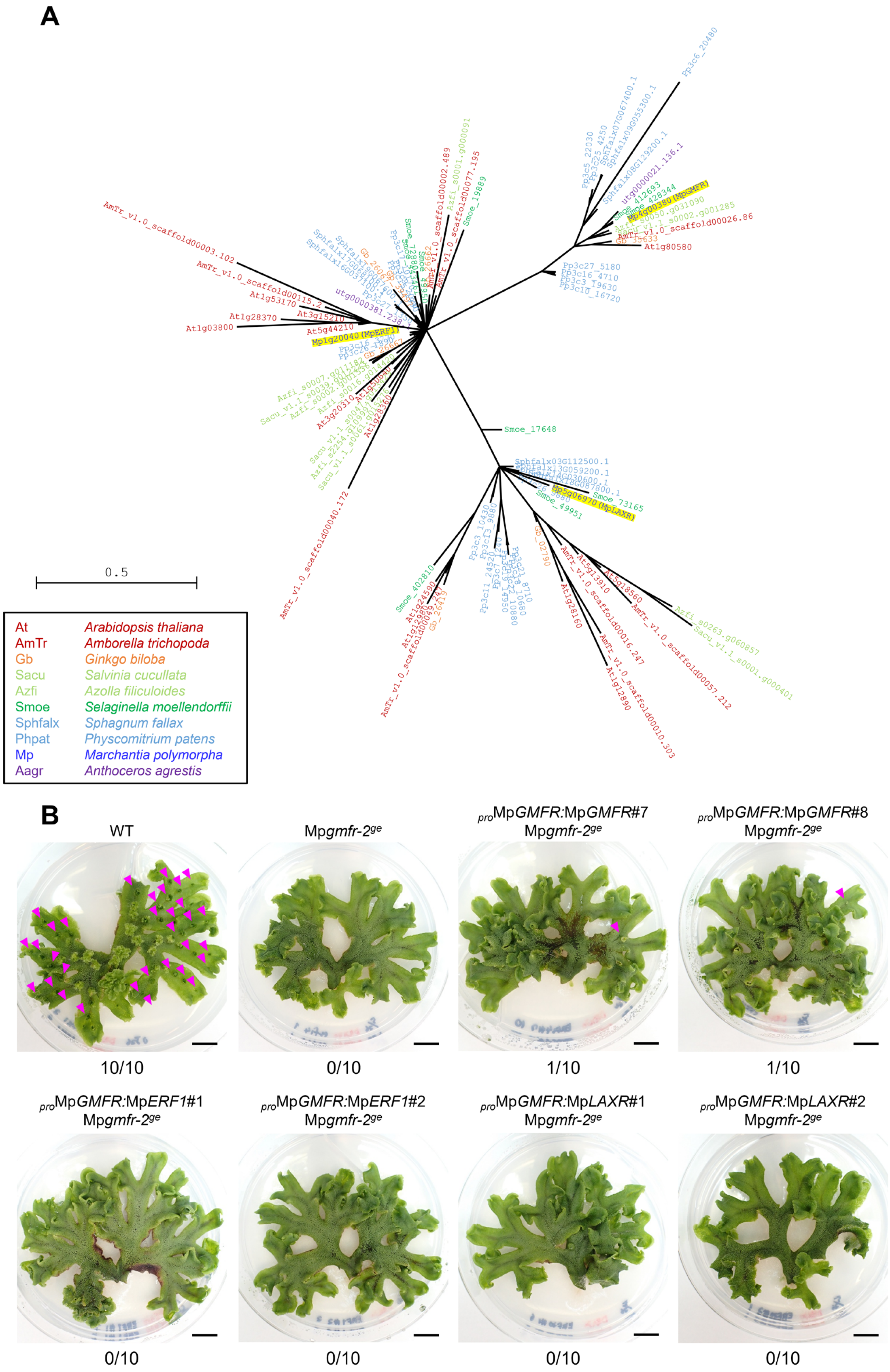

Figure S2. Analysis of ERF-VIII genes. Related to Figure 1.
(**A**) Molecular phylogenetic tree of class VIII of AP2/ERF family among land plant species, generated with a Bayesian method based on the conserved AP2 domain. (**B**) Complementation test of Mp*gmfr*^{ge} by Mp*GMFR-GR*, Mp*ERF1-GR* and Mp*LAXR-GR* driven under Mp*GMFR* promoter. The frequency of plants containing at least one gemma cup is indicated below each panel (n=10). Arrowheads indicate gemma cups. Scale bars, 1 cm.

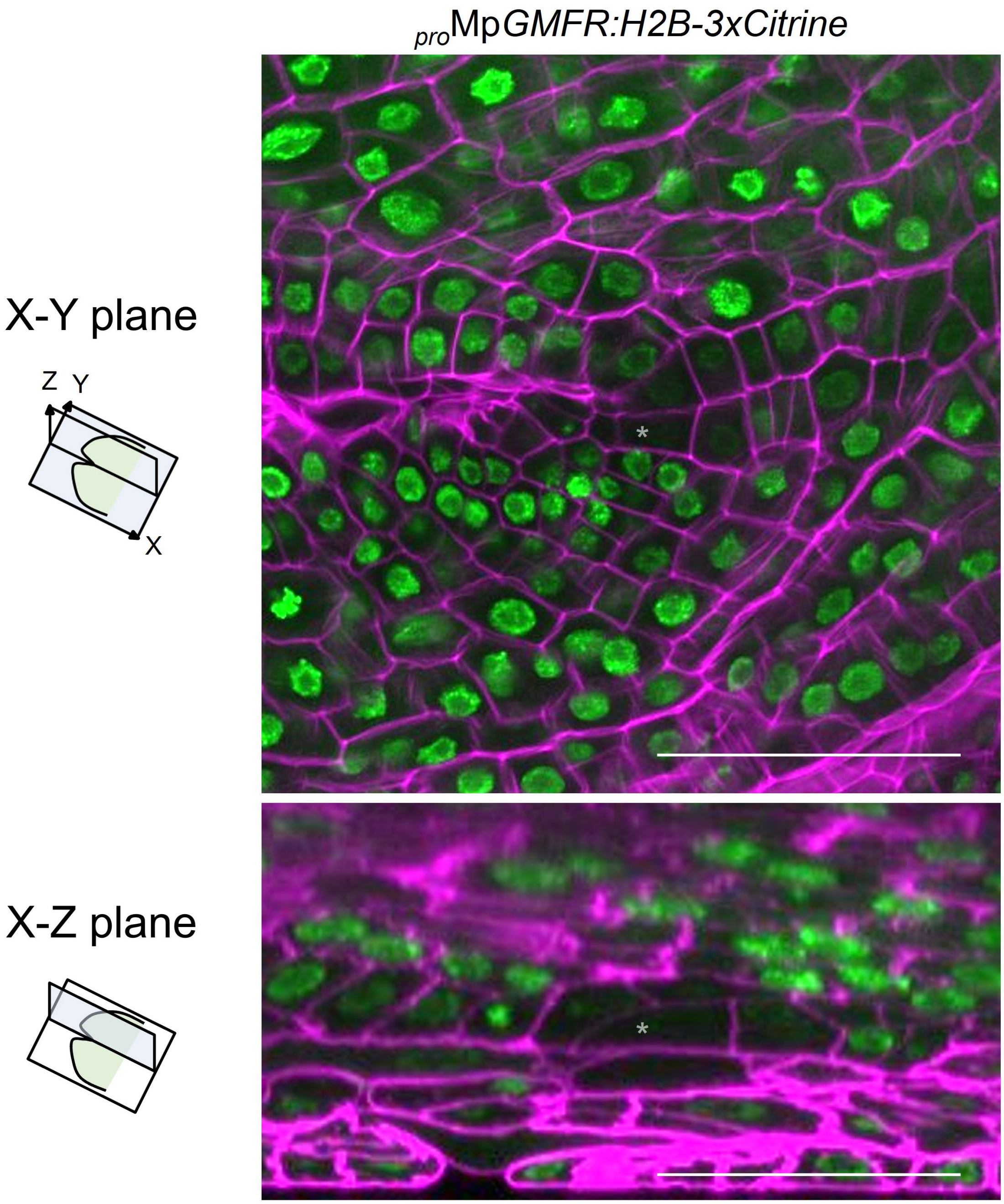

Figure S3. Expression patterns of Mp*GMFR* around the stem cell zone. Related to Figure 2.
Confocal imaging of the apical notch of $_{pro}$Mp*GMFR:H2B-3xCitrine* in 23-day-old plant. A single X-Y plane (top) and a reconstructed X-Z orthogonal view (bottom) derived from Z-stack images are indicated. Cell walls were stained with SCRI Renaissance 2200 (SR2200). Asterisks indicate a (sub)apical cell observed in the X-Y and X-Z planes. Scale bars, 50 µm.

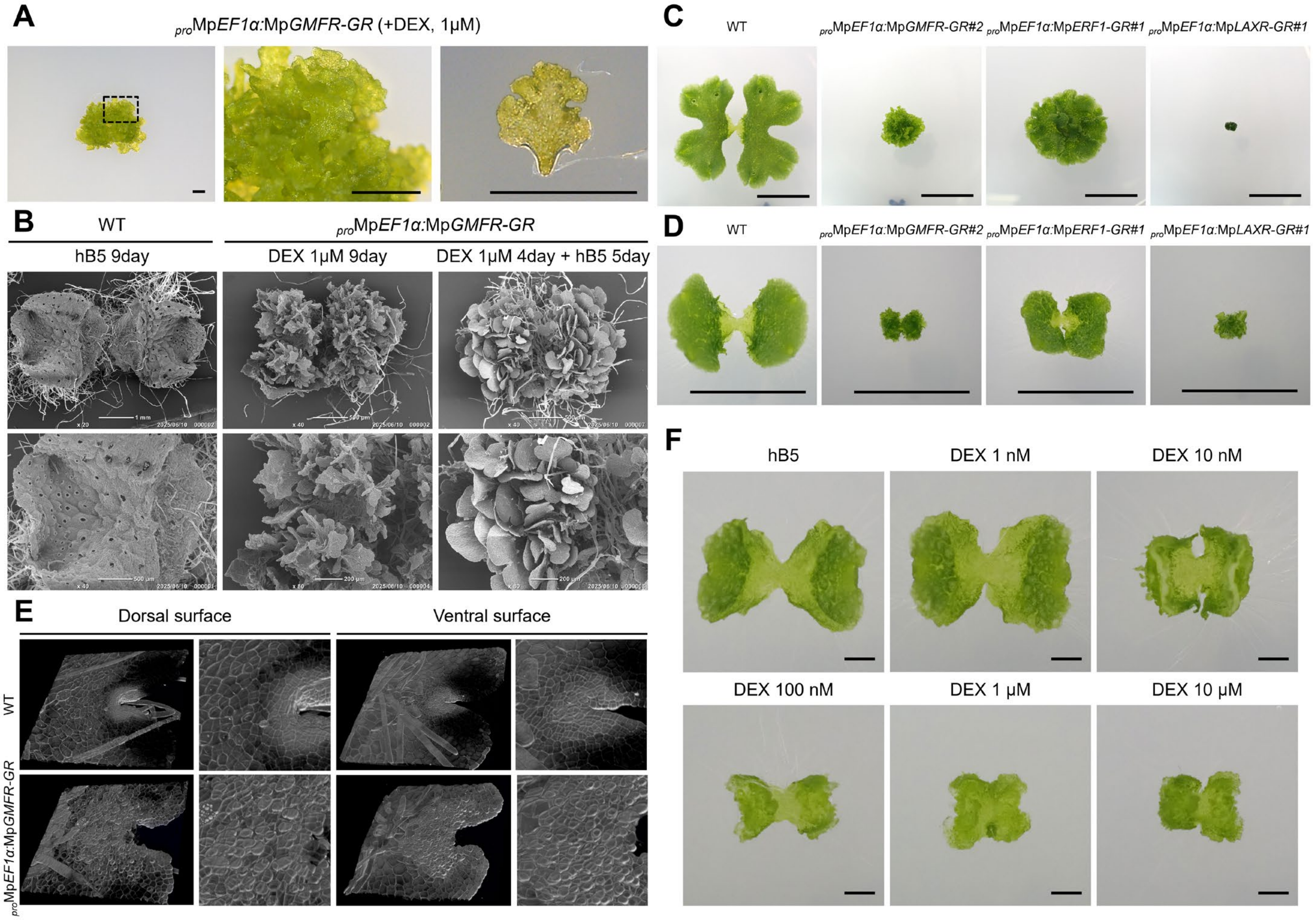

Figure S4. Overexpression phenotypes of Mp*GMFR* and its homologs. Related to Figures 3–5.
(**A**) Morphology of $_{pro}$Mp*EF1α:*Mp*GMFR-GR* plant grown on 1 μM DEX-containing medium for 9 days. The middle panel shows a close-up of the inset from the left panel. The right panel shows a magnified view of the tissue excised from the plant shown in the middle panel. Scale bars, 1 mm. (**B**) SEM images of the surface of 9-day-old thalli. Genotypes and growth conditions are indicated above the panels. Scale bar lengths are indicated in each panel. (**C** and **D**) Effects of inducible overexpression of Mp*GMFR*, Mp*ERF1* and Mp*LAXR*/Mp*ERF20*. Overall morphology of plants grown on 1 μM DEX-containing medium for 14 days (**C**) and those grown on 1 μM DEX-containing medium for 4 days followed by culture on DEX-free medium for 5 days (**D**). Scale bars, 1 cm. (**E**) Dorsal and ventral surfaces are shown from the 3D-reconstructed images of the apical notches in 3-day-old wild-type and $_{pro}$Mp*EF1α:*Mp*GMFR-GR* gemmalings. Right panels show magnified view of the areas indicated by dashed boxes in left panels. Cell walls were stained with SR2200. (**F**) Morphology of 7-day-old $_{pro}$Mp*EF1α:*Mp*GMFR-GR* plants grown from gemmae on media supplemented with indicated concentrations of DEX. Scale bars, 1 mm.

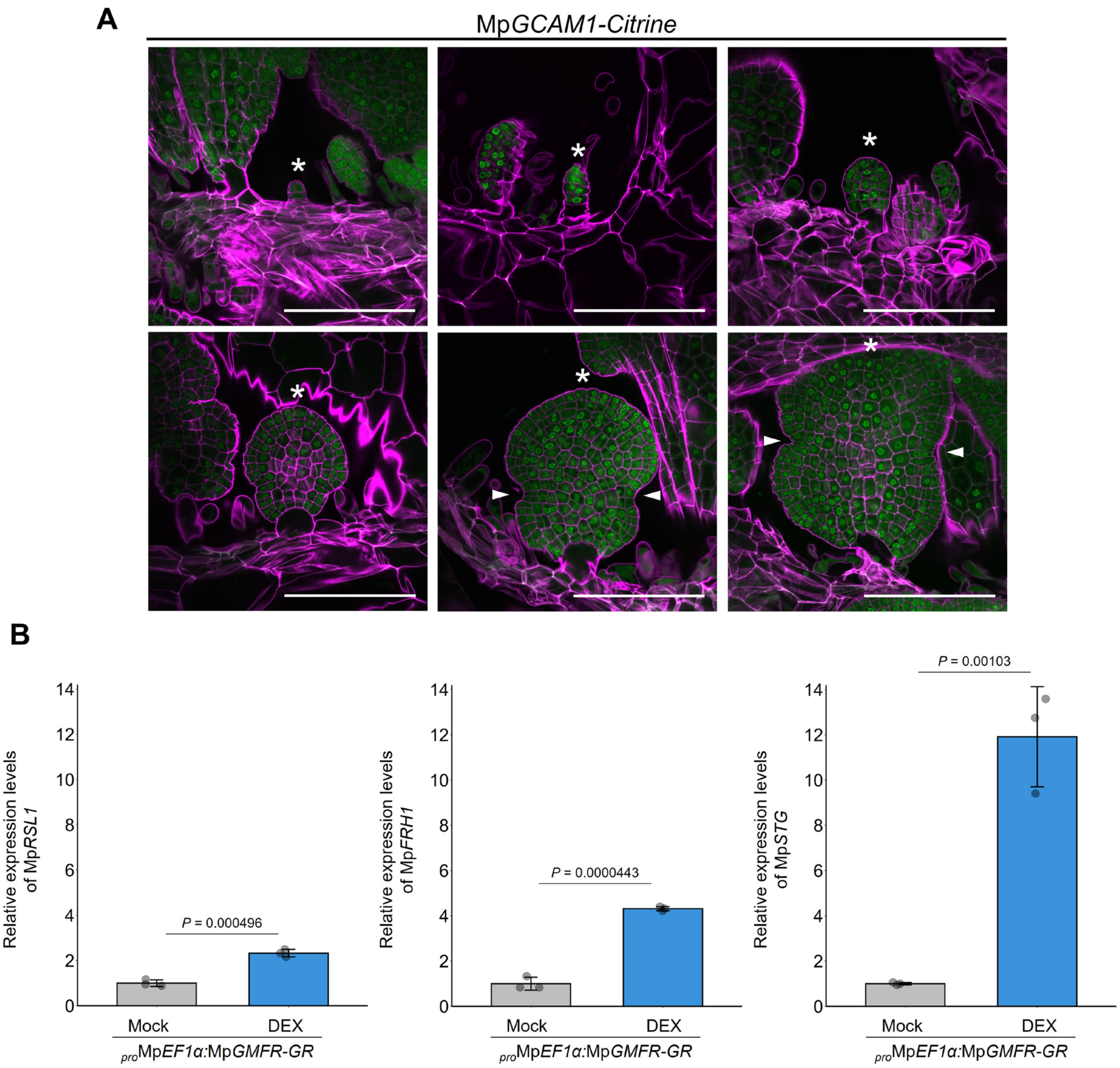

Figure S5. Expression analysis of known genes for gemma development. Related to Figures 2 and 6.
(**A**) Confocal imaging of Mp*GCAM1-Citrine* plants in developing gemmae in 14-day-old plants. Asterisks indicate developing gemmae. Arrowheads indicate apical notches. Cell walls were stained with SR2200. Scale bars, 100 μm. (**B**) Relative expression levels of Mp*RSL1,* Mp*FRH1* and Mp*STG* in $_{pro}$Mp*EF1α*:Mp*GMFR-GR* plants grown with mock or 1 μM DEX-containing medium for 4 days. Data are represented by mean and SD (bars) with individual data points (dots). Statistical significance was determined by Student's *t*-test. *P*-value is indicated above each pair of bars.

A

Mp*GCAM1*/Mp*6g04830*

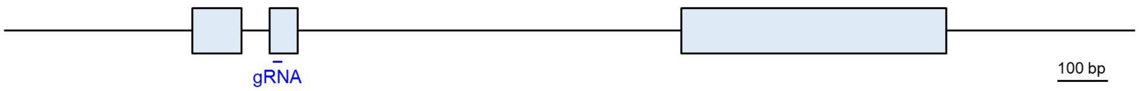


```
WT           GGTCGTCTTTTGTACAGGTCTGAAGCGC-TGCGGAAAGAGCTGC
Mpgcam1ge    GGTCGTCTTTTGTACAGGTCTGAAGCGCCTGCGGAAAGAGCTGC
```

B

```
WT
MQPLPTKQPSAATFVAVIFLALVAPFVAMGRAPCCDKANVKKGPWSPEEDAKLKSFIEHNGTGGNWITLPSKAGLKR
CGKSCRLRWINYLRPDIKHGSFTEEEEKTIYRLHAQIGSRWSLIAAQLPGRTDNDIKNYWNTRLKKKLLERASNMWC
GRPHHSFQIYPMNQPANDQMSNAVGREGLLNSQFLHAQAYYQYVQQQQPQYYLPQQQQQPQPIQDPSQTVQLPTVLQ
HQLHMRSIKNELVDEHQQQQAPTGGVVESERLRDIESSLRSIVDESSSRTFDARRAFQRLHCLSNRLPANGNPMSPA
AVATSHHNTSPTSSCSNLSSDRNSFGVQQIADSAFSDSGYTMYEDPAAREPSTTMLNTSILGSASQQQVASLPDTST
PESLLSTSRFEDGVFDDLSMYNRSQDLSEVAVVDRALGTGGVKEESHQASGGGSADWWANMDLVAPPGPLNQKSTVL
AANSMMRWSIPTPRCGAENIYNSPQAQLTSQSHGTQPLDGMNSYSDSFLSKLIS*
Mpgcam1ge
MQPLPTKQPSAATFVAVIFLALVAPFVAMGRAPCCDKANVKKGPWSPEEDAKLKSFIEHNGTGGNWITLPSKAGLKR
LRKELQTPLDQLFASRHQTWKLH*
```

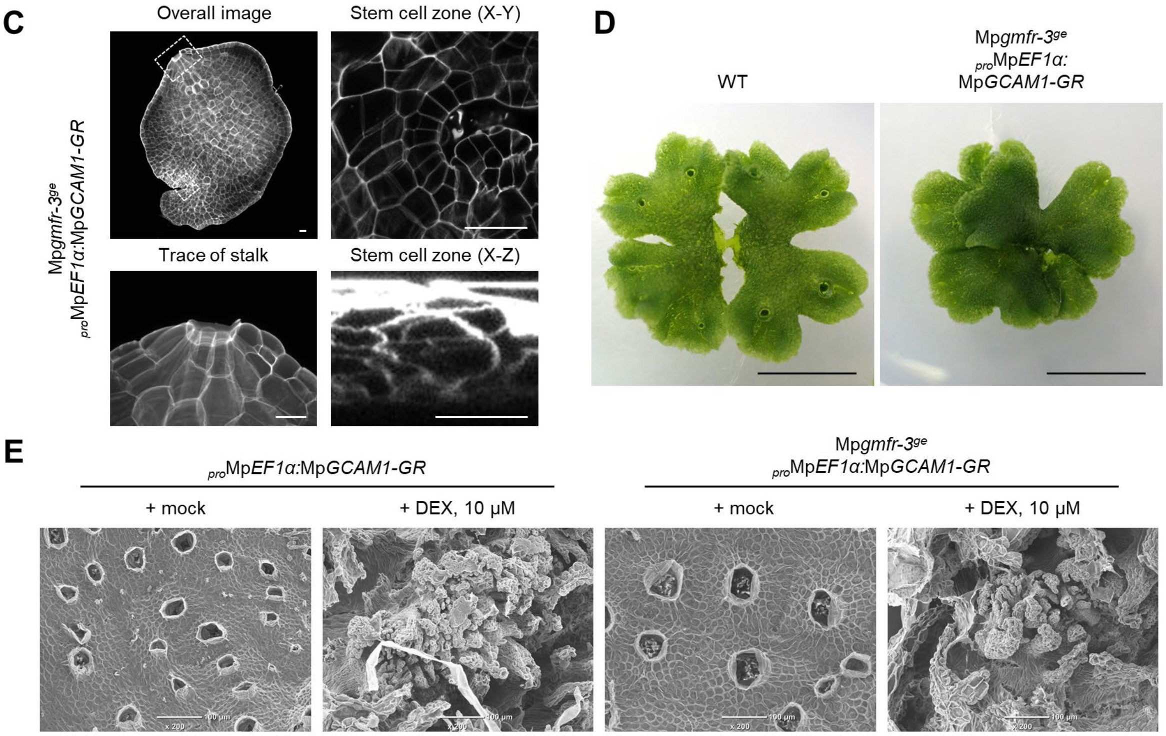

Figure S6. Functional analysis of Mp*GCAM1*. Related to Figure 6.
(**A**) Structure of Mp*GCAM1*/Mp6g04830 locus with the position of designed guide RNA (gRNA). Exons are shown as boxes. Genotyping of a genome editing allele is indicated below. gRNA sequence is in blue. An inserted base is in red. (**B**) Deduced MpGMFR protein sequences for WT and mutant. The sequence different from WT is in red. An asterisk indicates translational termination. (**C**) Confocal imaging of a gemma formed in Mp*gmfr-3*^{ge} $_{pro}$Mp*EF1α:*Mp*GCAM1-GR* plant by DEX treatment (top-left), containing a stem cell zone and a trace of stalk indicated in dashed boxes. The stem cell zone is shown in a single X-Y plane (top-right) and a reconstructed X-Z orthogonal view (bottom-right) derived from Z-stack images. The trace of stalk is shown in a 3D-reconstructed view (bottom-left). Scale bars, 25 µm. (**D**) Sixteen-day-old thalli grown from gemmae of WT and Mp*gmfr-3*^{ge} $_{pro}$Mp*EF1α:*Mp*GCAM1-GR*. Scale bars, 1 mm. (**E**) SEM images of the thallus surface of 14-day-old plants grown from explants including an apical notch on mock or 10 µM DEX-containing medium. Scale bars, 100 µm.

Primer Names	Sequence (5' to 3')	Purpose
**MpGMFRg_108F_A**	ctcgGCTGGCTCGACGAGCGTAGC	Genome editing
**MpGMFRg_108F_B**	aaacGCTACGCTCGTCGAGCCAGC	Genome editing
**MpGMFR_CDS_341R**	GACTTCAGATGGTCCTCCTCGAG	Genotyping
**MpGCAM1g_363f_A**	ctcgTGTACAGGTCTGAAGCGCTG	Genome editing
**MpGCAM1g_363f_B**	aaacCAGCGCTTCAGACCTGTACA	Genome editing
**MpGCAM1_CDS_617R**	GTAAACCCTACAAATGCGAGCAG	Genotyping
**MpGMFR_amiR_F**	cactTTTTCCCGTTTAATTGGGCCCACTGAGGAGCTCCTCAGAGACCTT GACAGGCTCCGTAGCGGGCCCGATTAAGCGGGGAAA	Artificial microRNA
**MpGMFR_amiR_R**	ctccTTTCCCCGCTTAATCGGGCCCGCTACGGAGCCTGTCAAGGTCTCT GAGGAGCTCCTCAGTGGGCCCAATTAAACGGGAAAA	Artificial microRNA
**MpGMFR_prom_F**	caccGCAGGGAGATTAATGGACAGGCG	pENTR cloning
**MpGMFR_prom_R**	GGATACTCCTATCGAAGAAACGACCG	pENTR cloning
**MpGMFR_CDS_F**	gcaggctccaccatgATGGCCTTGCATGATGTCCTC	pENTR cloning
**MpGMFR_CDS_R_+stop**	gtctagatatctcgaTCACAGACTGTAGGATCGCCATTG	pENTR cloning
**MpGMFR_CDS_R_-stop**	gtctagatatctcgaCAGACTGTAGGATCGCCATTGTTG	pENTR cloning
**MpGMFR_prom_F_InFusion_XbaI**	caagcttgggtctagaTTTAGCGACCGTGAAGAGGTGC	Insertion into Gateway Vector
**MpGMFR_prom_R_InFusion_XbaI**	tgttgataactctagaCTTCTAATCTTAACCGTGATCTTCTCTTCG	Insertion into Gateway Vector
**MpGMFR_CDS_gRNAres_F**	CTGGCTCGACGAGCGGAGTTGGAGATCTTGCGC	pENTR cloning
**MpGMFR_CDS_gRNAres_R**	GCGCAAGATCTCCAACTCCGCTCGTCGAGCCAG	pENTR cloning
**MpERF1_CDS_F**	gcaggctccaccatgATGGCTTTGAGGGACGAAGAGC	pENTR cloning
**MpERF1_CDS_R_-stop**	gtctagatatctcgaGGCCCGACTGCACAATGG	pENTR cloning
**MpLAXR_CDS_F**	gcaggctccaccatgATGGTGGGGAGGAAGCTGG	pENTR cloning
**MpLAXR_CDS_R_-stop**	gtctagatatctcgaCATGGGAACTAAAAGAAGAG	pENTR cloning
**MpGMFR_rt_F**	TGTATCGGCTGCCATGACTCC	RT-qPCR
**MpGMFR_rt_R**	AGCCGCAGACACTGGGATTG	RT-qPCR
**MpGCAM1_rt_F**	TGGATCAATTATTTGCGTCCCGAC	RT-qPCR
**MpGCAM1_rt_R**	ACCATCTGCTGCCAATTTGAGC	RT-qPCR
**MpRSL1_rt_F**	AGATGAGTCTGGGGCAACC	RT-qPCR
**MpRSL1_rt_R**	CGCTTTAGAGTGGTAGGCGC	RT-qPCR
**MpFRH1_rt_F**	GGGCTGCAGCACAAATCTAGATTC	RT-qPCR
**MpFRH1_rt_R**	GTTCACTGATGCACCGAACTTGG	RT-qPCR
**MpSTG_rt_F**	AACTCGTCCAAGATCACCATCC	RT-qPCR
**MpSTG_rt_R**	CAAGAGAGTAGATGCAGACGCT	RT-qPCR
**MpAPT_rt_F**	CGTCGTTGTTGGAATTGAAGC	RT-qPCR
**MpAPT_rt_R**	TTCTTGGGCTTTCGTTGAGG	RT-qPCR

Table S1. Primers. Related to STAR methods.
Small letters denote additional sequences for ligation, cloning and In-Fusion reaction.
