# Supplementary material for: Initiation of asexual reproduction by the AP2/ERF gene GEMMIFER in Marchantia polymorpha
Source: Curr Biol. 2026 May 4;36(9):2413–2423.e4. doi: 10.1016/j.cub.2026.03.083 (PMC13386412; doi:10.1016/j.cub.2026.03.083)
Supplement: Document S2. Article plus supplemental information [file mmc3.pdf]

Current Biology

Article

Initiation of asexual reproduction by the AP2/ERF gene *GEMMIFER* in *Marchantia polymorpha*

Graphical abstract

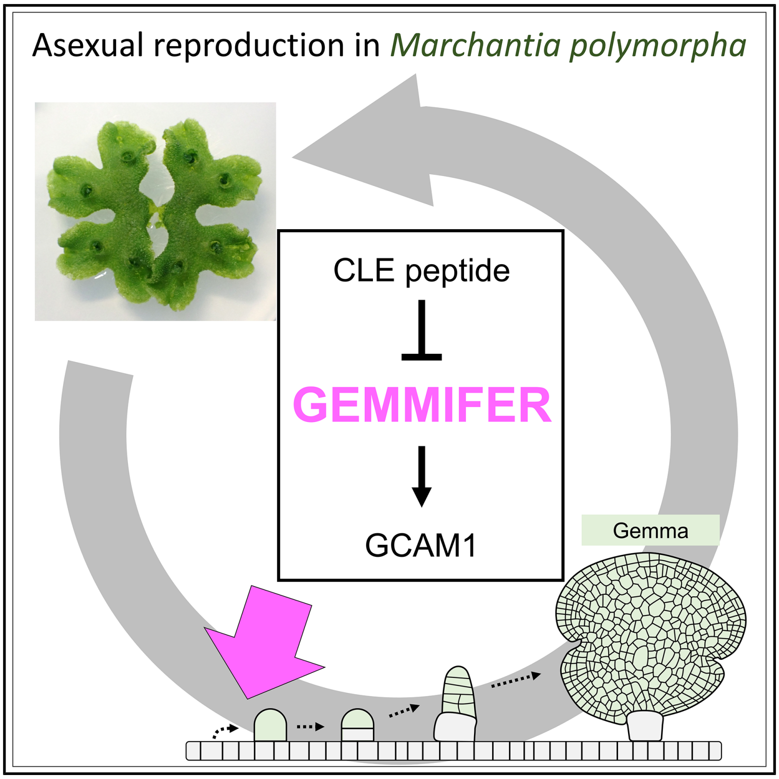

Authors

Go Takahashi, Saori Yamaya, Facundo Romani, ..., Tomohiro Kiyosue, Jim Haseloff, Yuki Hirakawa

Correspondence

yuki-hirakawa@hiroshima-u.ac.jp

In brief

Takahashi et al. show that the AP2/ERF gene *GEMMIFER* acts as a master regulator for the initiation of asexual propagule formation in the liverwort *Marchantia polymorpha*. Transient activation of the GEMMIFER protein induces the ectopic formation of propagules that can develop into new individuals after detachment from the parental plant.

Highlights

- Suppression of *GMFR* function results in the loss of gemma and gemma cup
- Transient activation of GMFR induces gemma and gemma cup formation at the meristem
- *GMFR* is expressed in the meristem and during early gemma development
- *GMFR* activates *GCAM1* expression as a key effector for gemma initiation

Takahashi et al., 2026, Current Biology *36*, 2413–2423
May 4, 2026 © 2026 The Authors. Published by Elsevier Inc.
https://doi.org/10.1016/j.cub.2026.03.083

CellPress

Current Biology

CellPress
OPEN ACCESS

Article

Initiation of asexual reproduction by the AP2/ERF gene *GEMMIFER* in *Marchantia polymorpha*

Go Takahashi,[1] Saori Yamaya,[2] Facundo Romani,[3] Ignacy Bonter,[3] Kimitsune Ishizaki,[4] Masaki Shimamura,[1] Tomohiro Kiyosue,[2] Jim Haseloff,[3] and Yuki Hirakawa[1,5,*]
[1]Graduate School of Integrated Sciences for Life, Hiroshima University, 1-3-1 Kagamiyama, Higashi Hiroshima, Hiroshima 739 8526, Japan
[2]Department of Life Science, Graduate School of Science, Gakushuin University, 1-5-1 Mejiro, Toshima-ku, Tokyo 171 8588, Japan
[3]Department of Plant Sciences, University of Cambridge, Cambridge CB2 3EA, UK
[4]Graduate School of Science, Kobe University, 1-1 Rokkodai, Kobe 657 8501, Japan
[5]Lead contact
*Correspondence: yuki-hirakawa@hiroshima-u.ac.jp
https://doi.org/10.1016/j.cub.2026.03.083

SUMMARY

Plants can propagate their own clones through asexual reproduction. Genetic and hormonal factors regulating asexual reproduction have begun to be elucidated in the liverwort *Marchantia polymorpha*, which produces asexual propagules called gemmae within the gemma cups. Here, we report an APETALA2/ETHYLENE RESPONSE FACTOR (AP2/ERF) family gene, *GEMMIFER* (Mp*GMFR*), as a key regulator of asexual reproduction in *M. polymorpha*. Suppression of Mp*GMFR* function using genome editing and artificial microRNA (amiRNA) results in the loss of gemma and gemma cup formation. In contrast, activation of MpGMFR function using a dexamethasone-inducible system promotes gemma and/or gemma cup formation, depending on the induction conditions. Notably, transient activation of MpGMFR induces gemma initial cells at the meristem, which develop into mature gemmae that are capable of reproducing as new individuals after detachment. Mp*GMFR* expression was detected from the meristem through to the early stages of gemma development including gemma cup floor cells, which precedes the expression of *GEMMA CUP-ASSOCIATED MYB1* (Mp*GCAM1*) in the early gemma development. Expression of Mp*GCAM1* was promoted by Mp*GMFR*, and overexpression of Mp*GCAM1* partially restored gemma and gemma cup formation in the Mp*gmfr* mutant. Taken together, Mp*GMFR* acts as a master regulator for the initiation of gemma and gemma cup development by activating Mp*GCAM1* as a key downstream effector. We anticipate this finding to serve as a foundation for studying the evolution of extra meristem formation in plant bodies, which may have made a significant contribution to the prosperity of plants on land.

INTRODUCTION

Plants have the remarkable ability to clone new individuals from their own bodies. This process, called asexual reproduction, occurs at various structures such as, adventitious shoots, bulbils, tubers, and rhizome buds.[1] Bryophytes often reproduce asexually through the dispersal of propagules called gemmae, with species-specific morphology.[2,3] In the liverwort *Marchantia polymorpha*, discoid-shaped gemmae are produced in specialized cup-shaped structures that are called gemma cups, which are generated periodically along the dorsal midrib of its thalloid body.[4] Recent studies on *M. polymorpha* have begun to elucidate the genetic and hormonal regulation of the gemma and gemma cup development.[5] An R2R3-MYB transcription factor, GEMMA CUP-ASSOCIATED MYB1 (MpGCAM1), has been identified as a gene that is highly expressed in gemma cups.[6] Molecular genetic analysis has shown that Mp*GCAM1* is required for the formation of gemma cups and gemmae, acting through the control of cell differentiation. Cytokinin signaling promotes the formation of gemma cups through upregulation of Mp*GCAM1* expression.[7,8] KARRIKIN INSENSITIVE2 (KAI2)-dependent signaling promotes gemma cup formation by upregulating the expression of a cytokinin biosynthesis enzyme, LONELY GUY (MpLOG), leading to the upregulation of Mp*GCAM1* expression.[9,10] Another R2R3-MYB transcription factor, SHOT GLASS (MpSTG), regulates the shape of the gemma cup and gemma development.[11] In addition, several genes are reported to be required for morphogenesis during early gemma development. Mp*ROOT HAIR DEFECTIVE SIX-LIKE1* (Mp*RSL1*) transcription factor gene, targeted by the microRNA *FEW RHIZOIDS1* (Mp*FRH1*), is required for cellular outgrowth at the floor of gemma cup, which later develops into gemmae.[12,13] The single-copy *RHO of Plant* (Mp*ROP*) gene and its regulatory factors are essential for the morphogenesis of various tissues and organs, including gemmae and gemma cups.[14–17] Signaling by the plant hormones auxin, ethylene, and jasmonate affects the morphology of gemmae.[18–20] Although these studies have identified a number of genes regulating gemma development, the key genes that control the initiation of the gemma cell lineage remain unknown.

We have previously shown that MpCLAVATA3/EMBRYO SURROUNDING REGION-related 2 (MpCLE2) peptide signaling

© 2026 The Authors. Published by Elsevier Inc.
This is an open access article under the CC BY license (http://creativecommons.org/licenses/by/4.0/).

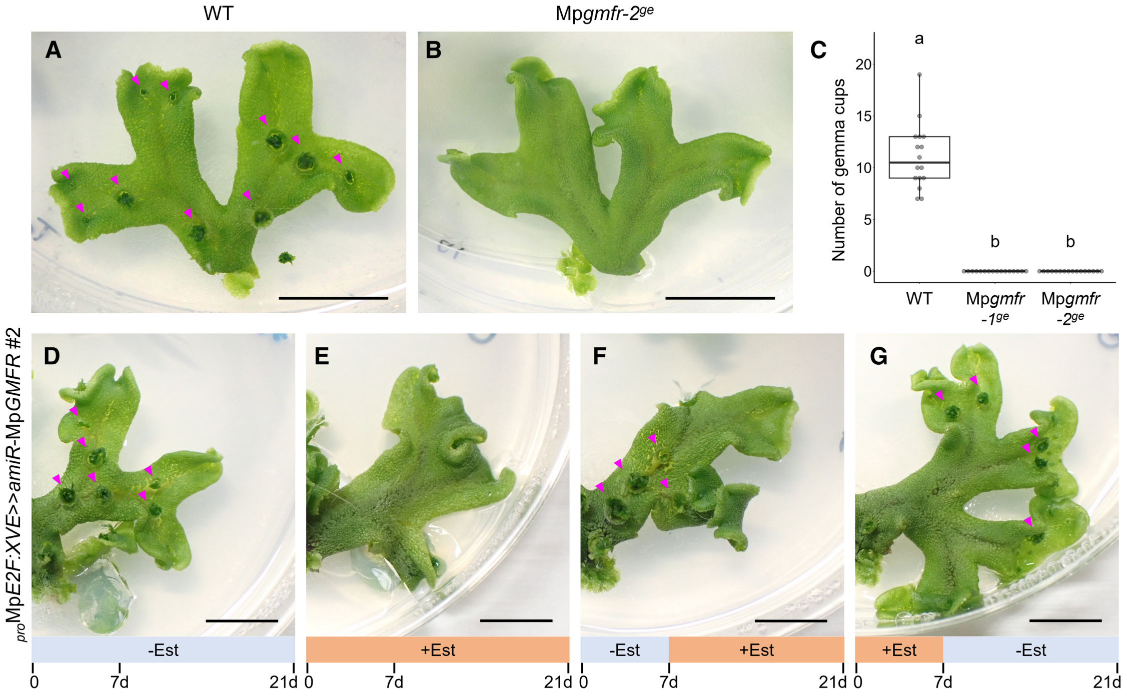

Figure 1. MpGMFR is essential for the formation of gemma cups and gemmae

(A and B) 2-week-old wild type (A) and Mp*gmfr-2*^{ge} (B) plants grown from explants containing an apical notch. Scale bars, 1 cm.

(C) Quantification of the number of gemma cups (n = 16).

(D–G) Effects of Mp*GMFR* knockdown on gemma cup formation in $_{pro}$Mp*E2F:XVE* >> *amiR*-Mp*GMFR*. Expression of *amiR*-Mp*GMFR* was induced by 5 μM β-estradiol (Est) during different periods within the 21 days as indicated below the panels. Scale bars, 1 cm.

In (A), (B), and (D)–(G), arrowheads indicate gemma cups. In (C), the boxes show the median and interquartile range (IQR), and the whiskers extend to 1.5× IQR. Individual data points are plotted as dots. Statistical significance was determined by two-way ANOVA with Tukey's post-hoc test; means sharing the same superscript letters are not significantly different, $p < 0.05$.

See also Figures S1 and S2.

negatively regulates the formation of gemma cups, in addition to its function in stem cell identity in the meristem located in the apical notch.[21–23] In a transcriptome analysis, we have identified several differentially expressed transcription factor (DETF) genes in Mp*CLE2* gain-of-function transgenic lines. Among them, *JINGASA* (Mp*JIN*/Mp*NAC6*) affects stem cell fate in the meristem by promoting periclinal cell division.[24] In this study, we report that another DETF gene, *GEMMIFER* (Mp*GMFR*)/Mp*ERF14*, plays a key role in the initiation of asexual reproduction. This finding provides a molecular clue to understanding how plant cell fate is regulated in asexual reproduction.

RESULTS

Mp*GMFR* is essential for the formation of gemma cups and gemmae

To understand the function of Mp*GMFR*, we generated loss-of-function alleles using the CRISPR-Cas9 genome editing. Two independent frameshift alleles (Mp*gmfr-1*^{ge} and Mp*gmfr-2*^{ge}) were obtained (Figures S1A and S1B), and both resulted in the complete loss of gemma cup and gemma formation. For the quantification, we observed a 2-week-old thallus developed from an explant containing an apical notch. In the wild type, all examined plants formed gemma cups on the dorsal surface (Figure 1A). In contrast, none of the Mp*gmfr-2*^{ge} plants formed gemma cups and gemmae (Figures 1B and 1C). This Mp*gmfr*^{ge} phenotype was partially complemented by the introduction of a gRNA-resistant Mp*GMFR* expressed under its own promoter (Figures S1C and S1D). To further elucidate the function of Mp*GMFR*, we generated estrogen-inducible artificial microRNA (amiRNA) lines that target Mp*GMFR* mRNA using the XVE transactivation system ($_{pro}$Mp*E2F:XVE* >> *amiR*-Mp*GMFR*). In RT-qPCR assays, Mp*GMFR* mRNA levels were decreased in a β-estradiol-dependent manner in the $_{pro}$Mp*E2F:XVE* >> *amiR*-Mp*GMFR* plants compared with those in wild-type plants (Figure S1E). The $_{pro}$Mp*E2F:XVE* >> *amiR*-Mp*GMFR* plants grown for 3 weeks on β-estradiol-free medium formed gemma cups, but those on 5 μM β-estradiol-containing medium did not (Figures 1D and 1E). We transferred the 2-week-old plants from β-estradiol-free to β-estradiol-containing medium and grew them further for 1 week, or vice versa. The transferred plants lost the formation of gemma cups and gemmae in a β-estradiol-dependent, reversible manner (Figures 1F and 1G). Collectively, these data show that Mp*GMFR* is required for the formation of gemma cups and gemmae in *M. polymorpha*.

Molecular phylogenetic analysis has shown that *M. polymorpha* possesses three genes: Mp*ERF1* (Mp1g20040), Mp*ERF14/GMFR* (Mp4g00380), and Mp*ERF20/LOW-AUXIN RESPONSIVE* (*LAXR*) (Mp5g06970), in class VIII of the AP2/ERF (ERF-VIII) family.[25,26] A phylogenetic tree of ERF-VIII genes from various land plant

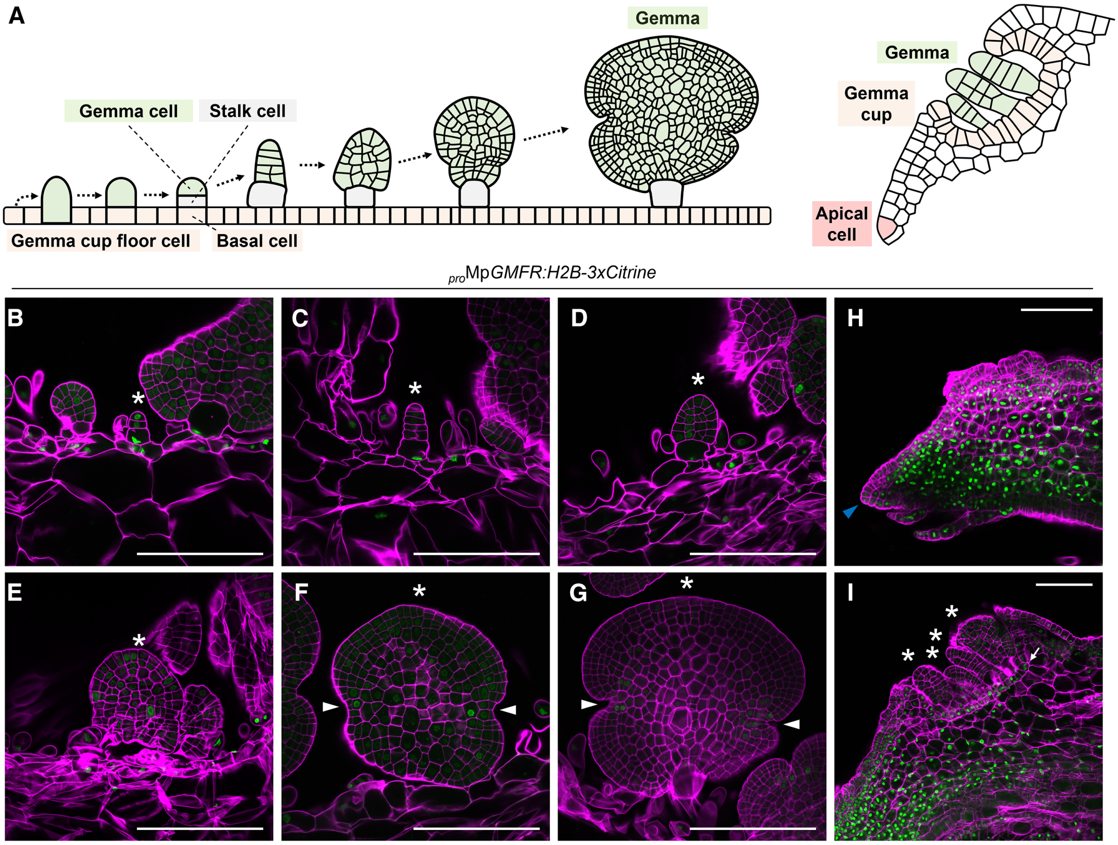

Figure 2. Expression patterns of Mp*GMFR* in developing gemma and gemma cup

(A) Schematic illustration of the early development of gemma (left) and developing gemma cup (right, modified from Barnes and Lang[28]).

(B–I) Confocal imaging of $_{pro}$Mp*GMFR:H2B-3xCitrine* in developing gemmae (B–G) in 14-day-old plants and cross-sections of thallus in 11-day-old (H) and 12-day-old (I) plants. Cell walls were stained with SCRI Renaissance 2200 (SR2200). Asterisks show developing gemmae. Blue and white arrowheads indicate a subapical cell and apical notches, respectively. An arrow indicates the layer of gemma cup floor cells. Scale bars, 100 µm.

See also Figures S3 and S5.

species (*Arabidopsis thaliana*, *Amborella trichopoda*, *Ginkgo biloba*, *Salvinia cucullata*, *Azolla filiculoides*, *Selaginella moellendorffii*, *Sphagnum fallax*, *Physcomitrium patens*, *Marchantia polymorpha,* and *Anthoceros agrestis*) suggests that ERF-VIII can be divided into three subgroups, each of which contains a single *M. polymorpha* gene (Figure S2A). To verify the functional redundancy among ERF-VIII genes in *M. polymorpha*, we conducted a complementation test of the Mp*gmfr*^{ge} phenotype by introducing the coding sequences of Mp*ERF1* and Mp*LAXR*, respectively, that are expressed under Mp*GMFR* promoter. Neither construct complemented the Mp*gmfr*^{ge} phenotype, suggesting that Mp*GMFR* plays an essential function in the gemma cup and gemma formation that cannot be replaced by Mp*ERF1* or Mp*ERF20/LAXR* (Figure S2B).

Transient activation of MpGMFR induces ectopic gemmae formation

In *M. polymorpha*, gemmae are generated from the floor cells at the bottom of the gemma cup. At the initiation of gemma development, a gemma cup floor cell protrudes from the epidermal surface and then transversely divides twice to form three cells: a gemma cell, a stalk cell, and a basal cell[27,28] (Figure 2A). While the stalk cell ceases division, the gemma cell continues to divide, developing into a discoid gemma with two bilaterally located apical meristematic notches. Thus, the gemma cell acts as the initial cell for the development of gemma.

To analyze the expression patterns of Mp*GMFR* during asexual reproduction, we observed $_{pro}$Mp*GMFR:H2B-3xCitrine* transcriptional fusion reporter lines in cross-sections of gemma cups cleared via the iTOMEI method,[29] using confocal laser scanning microscopy. The fluorescence signals of $_{pro}$Mp*GMFR:H2B-3xCitrine* were detected in the gemma cup floor cells (Figures 2B–G). In the initial stage of gemma development, the signals were also detected in both the gemma cell and the stalk cell (Figure 2B) but were lost in the gemma cell after several subsequent divisions (Figure 2C). At a later stage, the signals were scattered within the developing gemmae (Figures 2D and 2E). After the formation of meristematic notches at bilateral sites of the gemmae, the signals were restricted to cells near the notches (Figures 2F and 2G). This pattern is consistent with the promoter activity observed in gemmae (https://mpexpatdb.org/).[30] In the vertical longitudinal section of the apical notch of mature plants, the signals were broadly detected at the meristem,

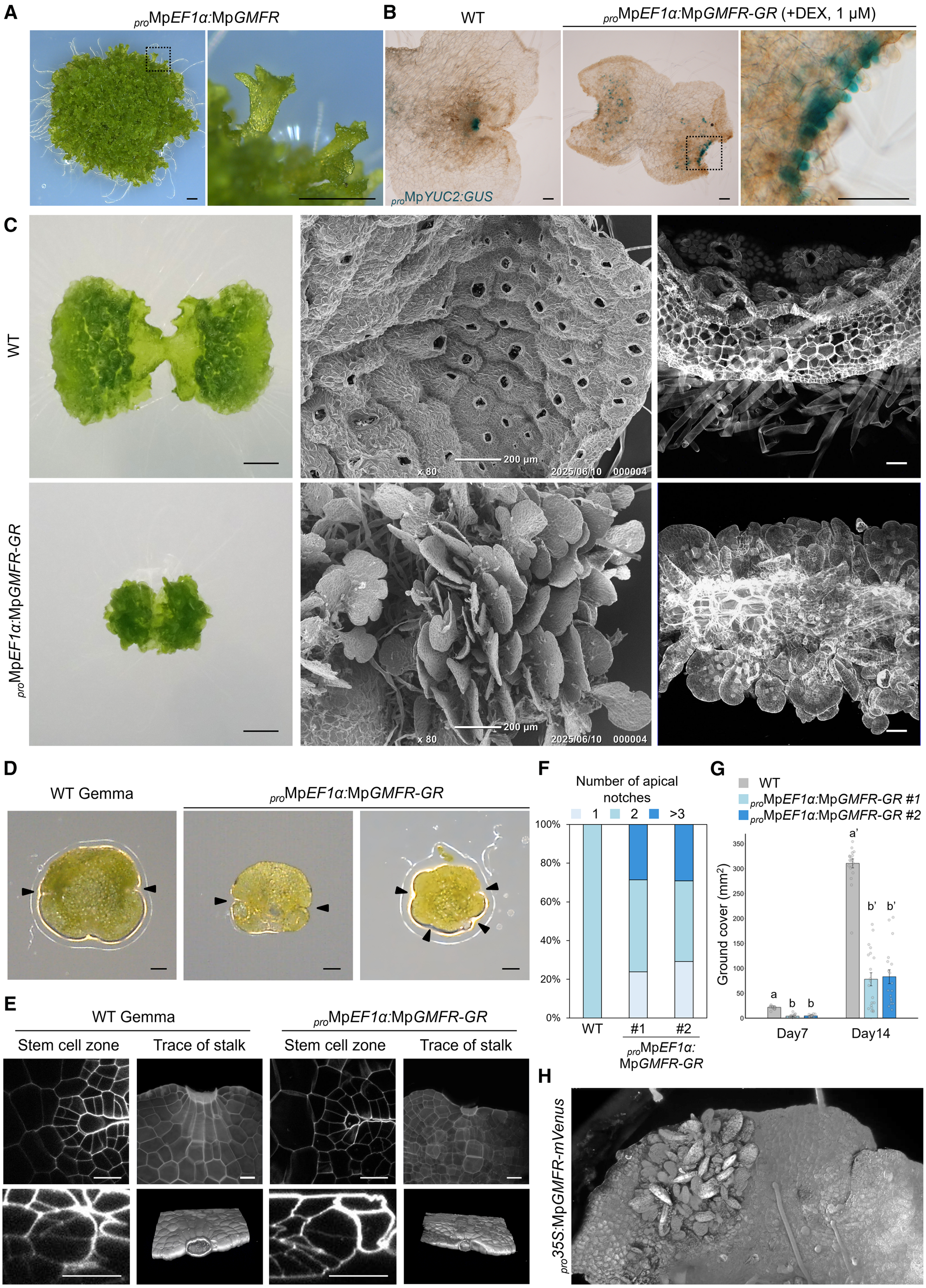

Figure 3. Transient activation of MpGMFR induces ectopic gemmae formation

(A) Morphology of $_{pro}$Mp*EF1α*:Mp*GMFR* plant. The right panel shows a magnification of the dashed box in the left panel. Scale bars, 1 mm.

(B) Morphology of apical notch with the $_{pro}$Mp*YUC2:*GUS marker in 4-day-old gemmalings. The right panel shows a magnification of the dashed box in the center panel. Scale bars, 100 μm.

(legend continued on next page)

CellPress
OPEN ACCESS

except for the central part near the apical/subapical cells (Figures 2H and S3). The signals were detected in the floor cells of the immature gemma cup but not in gemmae developing in the cup (Figure 2I). These data suggest that Mp*GMFR* expression is associated with the meristematic region, gemma cup floor cells, and the very early stages of gemma development.

To understand the function of Mp*GMFR*, we first generated overexpression lines using the constitutive Mp*EF1*α promoter. The $_{pro}$Mp*EF1*α*:*Mp*GMFR* plants result in ball-shaped structures composed of small immature thalli (Figure 3A). To analyze the short-term effects of overexpression, we generated inducible overexpression lines ($_{pro}$Mp*EF1*α*:*Mp*GMFR-GR*), in which MpGMFR proteins fused with glucocorticoid receptor (GR) were expected to be translocated into the nucleus by dexamethasone (DEX) treatment. In 1-μM-DEX-containing medium, 4-day-old $_{pro}$Mp*EF1*α*:*Mp*GMFR-GR* gemmalings formed protruding cells around the apical notch (Figure 3B). These cells can be stained by $_{pro}$Mp*YUC2:GUS* and have a morphology that is similar to the gemma cell in the initial stages of gemma development. However, 9-day-old $_{pro}$Mp*EF1*α*:*Mp*GMFR-GR* plants grown on the DEX-containing medium did not form gemmae and only formed ball-shaped structures composed of immature small thalli, phenocopying the constitutive overexpression lines (Figures S4A and S4B). Since Mp*GMFR* expression is suggested to be transiently active in the early development of gemmae (Figures 2A and 2B), we hypothesized that the continuous DEX induction of MpGMFR compromised the normal development of gemmae. To avoid this, 4-day-old $_{pro}$Mp*EF1*α*:*Mp*GMFR-GR* plants grown on DEX-containing medium were transferred to DEX-free medium for further growth. 5 days after the transfer, a number of gemmae were formed on both the dorsal and ventral sides of the thallus (Figure 3C). The induced gemmae can be detached with water and contain apical notches for further growth (Figure 3D). These notches contained apical and subapical cells, showing a cellular arrangement that is characteristic of a typical stem cell zone (Figure 3E). In contrast to wild-type gemmae, which possess two apical notches at bilateral sites, the number of apical notches varied from one to four among the individual induced gemmae (Figure 3F). In addition to the apical notches, each induced gemma had a trace of a stalk, with a cell arrangement similar to that of the wild type (Figure 3E). Although these gemmae were smaller than the wild type in overall size (ground cover area), they were viable and grew normally on the medium (Figures 3D and 3G). Consistently, 5-day-old $_{pro}$*35S*:Mp*GMFR-mVenus* gemmalings formed ectopic gemmae on the surface (Figure 3H). Activities of other ERF-VIII genes, Mp*ERF1* and Mp*LAXR*, were examined by generating $_{pro}$Mp*EF1*α*:*Mp*ERF1-GR* and $_{pro}$Mp*EF1*α*:*Mp*LAXR-GR* lines. In both cases, ectopic gemmae were not induced in contrast to $_{pro}$Mp*EF1*α*:*Mp*GMFR-GR* plants, supporting the notion that ERF-VIII genes do not exert redundant function in *M. polymorpha* (Figures S4C and S4D). Taken together, these results demonstrate that the transient induction of MpGMFR leads to the ectopic formation of gemmae that are capable of reproducing as new individuals.

To investigate the timing of gemma initiation after MpGMFR-GR induction, we observed apical notches of $_{pro}$Mp*EF1*α*:*Mp*GMFR-GR* plants grown for 1–4 days on 1 μM DEX-containing medium. On day 2, protruding cells that have undergone transverse division were observed at the apical notch (Figure 4A). The protruding cells have undergone a subsequent transverse division to produce a gemma cell and a stalk cell on day 3. On day 4, the gemma cells divided further, whereas the stalk cells did not divide, recapitulating the division patterns in the early development of wild-type gemmae. In some cases, the cell division patterns of the gemma cell were unusual compared with those in the wild type, which might be the cause of the malformation of gemmae (Figures 3D and 3F). In the 3D-reconstructed images of apical notches of the parent plants, protruding cells and early-developing gemmae were observed on both dorsal and ventral surfaces in $_{pro}$Mp*EF1*α*:*Mp*GMFR-GR* plants, whereas no gemmae were detected in the wild type (Figures 4B and S4E). These data indicate that Mp*GMFR* controls the initiation of gemma cell lineage in the meristem.

Weak induction of MpGMFR promotes gemma cup formation

To examine the concentration-dependent effects of MpGMFR, $_{pro}$Mp*EF1*α*:*Mp*GMFR-GR* plants were grown on growth media containing different concentrations of DEX (Figure S4F). In contrast to the 1 μM DEX treatment, which only induced the formation of gemma-like tissues, 10 nM DEX treatment led to the formation of semi-circular gemma cups near the apical notches, which contain gemma-like tissues inside, in 7-day-old plants (Figure 5A). The rim structure of the induced gemma

(C) Morphology of 9-day-old plants grown from gemmae. Wild-type plants were grown for 9 days on normal medium throughout. $_{pro}$Mp*EF1*α*:*Mp*GMFR-GR* plants were grown on 1-μM-DEX-containing medium for 4 days, then transferred to DEX-free medium and grown for an additional 5 days. Overall morphology (left), scanning electron microscopy (SEM) images of the thallus surface (center), and 3D-reconstruction of confocal images of the thallus cross-section (right) are shown. Scale bars, 1 mm (left), 200 μm (center), or 100 μm (right).

(D) Gemma morphology of wild-type (left) and $_{pro}$Mp*EF1*α*:*Mp*GMFR-GR* (right two panels) plants. Arrowheads indicate meristematic notches. Scale bars, 100 μm.

(E) Confocal imaging of the stem cell zone and the trace of stalk in wild-type (left) and $_{pro}$Mp*EF1*α*:*Mp*GMFR-GR* (right) gemmae. The stem cell zone is shown in a single X-Y plane (top) and a reconstructed X-Z orthogonal view (bottom) derived from z stack images. The traces of stalk are shown in a single X-Y plane (top) and a 3D-reconstructed view (bottom). Scale bars, 25 μm.

(F) Distribution of the number of apical notches per plant (n = 20–23).

(G) Ground cover area of 7- and 14-day-old plants grown from gemmae (n = 18–20).

(H) 3D-reconstructed view of a 5-day-old $_{pro}$*35S:*Mp*GMFR-mVenus* gemmaling.

In (G), data are represented by mean and SD (bars) with individual data points (dots). Statistical significance was determined by two-way ANOVA with Tukey's post-hoc test; means sharing the same superscript letters are not significantly different, $p < 0.05$.

In (C) and (E), cell walls were stained with SR2200.

See also Figure S4.

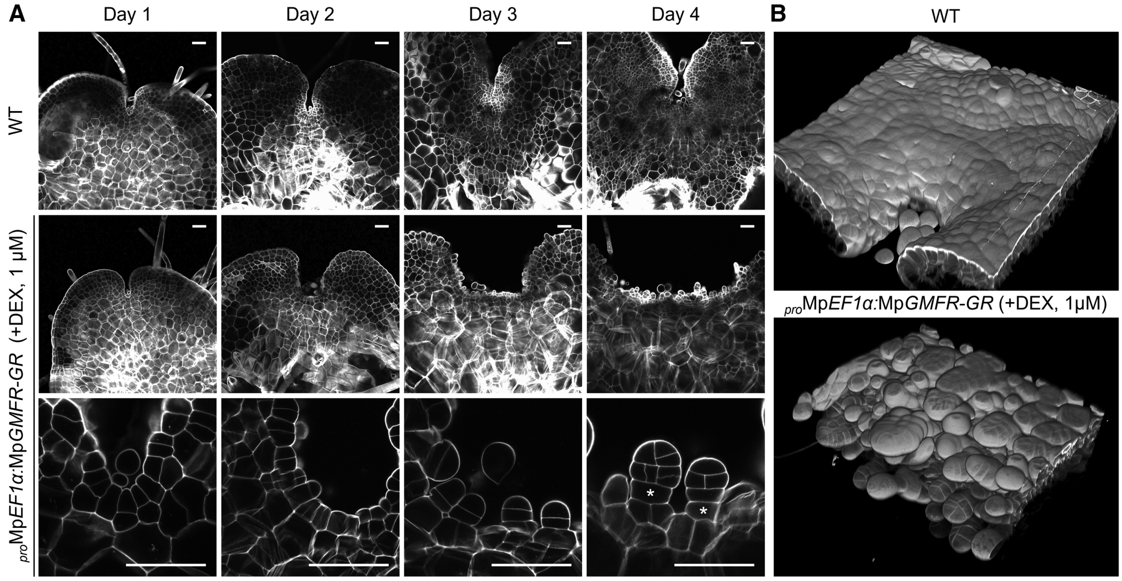

Figure 4. MpGMFR initiates the gemma cell lineage in the meristem

(A) Confocal images of the apical notches in wild-type and $_{pro}$Mp*EF1α*:Mp*GMFR-GR* plants grown with 1 μM DEX. Panels in the bottom row show high-magnification views of the apical notches shown in the middle row. Asterisks indicate stalk cells. Scale bars, 50 μm.

(B) 3D-reconstructed view of apical notches in 4-day-old plants.

In (A) and (B), cell walls were stained with SR2200.

See also Figure S4.

cups in $_{pro}$Mp*EF1α:*Mp*GMFR-GR* plants was similar to that of wild-type gemma cups (Figure 5B). These data suggest that Mp*GMFR* can also induce the formation of gemma cups.

To examine the functional relationship between Mp*CLE2* and Mp*GMFR* genes, we generated $_{pro}$Mp*EF1α:*Mp*GMFR-GR* lines in the background of a gain-of-function allele of Mp*CLE2*, $_{pro}$Mp*YUC2:*Mp*CLE2-2*.[21] In this background, overexpression of Mp*CLE2* resulted in reduced gemma cup formation, accompanied by decreased Mp*GMFR* expression.[22,24] For the phenotypic analysis, gemmae were first grown on DEX-free medium for 12 days, followed by an additional 8 days of culture on a 10 nM DEX-containing medium. As a result, two independent $_{pro}$Mp*EF1α:*Mp*GMFR-GR* lines showed a significant increase in the number of gemma cups compared with the background line (Figures 5C and 5D), suggesting that Mp*CLE2* overexpression phenotypes on gemma cup formation can be suppressed by restoring the reduced Mp*GMFR* expression.

Genetic interaction of Mp*GMFR* and Mp*GCAM1*

Loss-of-function alleles of Mp*GCAM1* are deficient in gemma cups and gemmae,[6] similar to those of Mp*GMFR*. To analyze the functional relationship between these genes, we compared their expression patterns during gemma development. While the Mp*GMFR* expression was detected in gemma cup floor cells and during early gemma development (Figure 2), the Mp*GCAM1-Citrine* knockin allele showed strong fluorescence throughout gemma development, but signals were barely detectable in gemma cup floor cells[6,11] (Figure S5A), suggesting that Mp*GMFR* expression precedes Mp*GCAM1* expression in the gemma development. We further examined the expression levels of each gene in the mutants of the other gene by RT-qPCR assays. In the 6-day-old plants grown from explants containing an apical notch, the Mp*GCAM1* mRNA level was decreased to approximately 4% in Mp*gmfr^{ge}* lines compared to wild type (Figure 6A). In the 4-day-old gemmalings of the $_{pro}$Mp*EF1α:*Mp*GMFR-GR* line grown with DEX, the Mp*GCAM1* mRNA level was increased to approximately 750% compared to mock treatment (Figure 6B). Similarly, the mRNA levels of Mp*RSL1*, Mp*FRH1,* and Mp*STG*, genes known to be required for gemmae formation, were increased by DEX treatment in 4-day-old gemmalings of the $_{pro}$Mp*EF1α:*Mp*GMFR-GR* line (Figure S5B). On the other hand, the Mp*GMFR* mRNA level was not significantly affected in the Mp*gcam1^{ko}* line[6] compared to wild type in 6-day-old plants grown from explants containing an apical notch (Figure 6C). In the 4-day-old gemmalings of an Mp*GCAM1-GR* overexpression line[6] grown with DEX, the Mp*GMFR* mRNA level decreased to approximately 74% compared to mock treatment (Figure 6D). These data suggest that Mp*GMFR* promotes Mp*GCAM1* expression while Mp*GCAM1* has little impact on Mp*GMFR* expression.

To further analyze the functional relationship, we introduced frameshift mutations in Mp*GCAM1* by using CRISPR-Cas9 genome editing in the $_{pro}$Mp*EF1α:*Mp*GMFR-GR* background (Figures S6A and S6B). As expected, gemma and gemma cup formation were completely lost in these lines, and we used one of them for further analysis. To analyze the effects on gemma cup formation, explants containing an apical notch were grown on 1 μM DEX-containing medium for 7 days, followed by an additional 7 days on DEX-free medium. Under these conditions, gemma-like tissues were formed in $_{pro}$Mp*EF1α:*Mp*GMFR-GR* plants. In contrast, such gemma-like tissues were not observed in the Mp*gcam1^{ge}* $_{pro}$Mp*EF1α:*Mp*GMFR-GR* line (Figure 6E). Also, we

CellPress
OPEN ACCESS

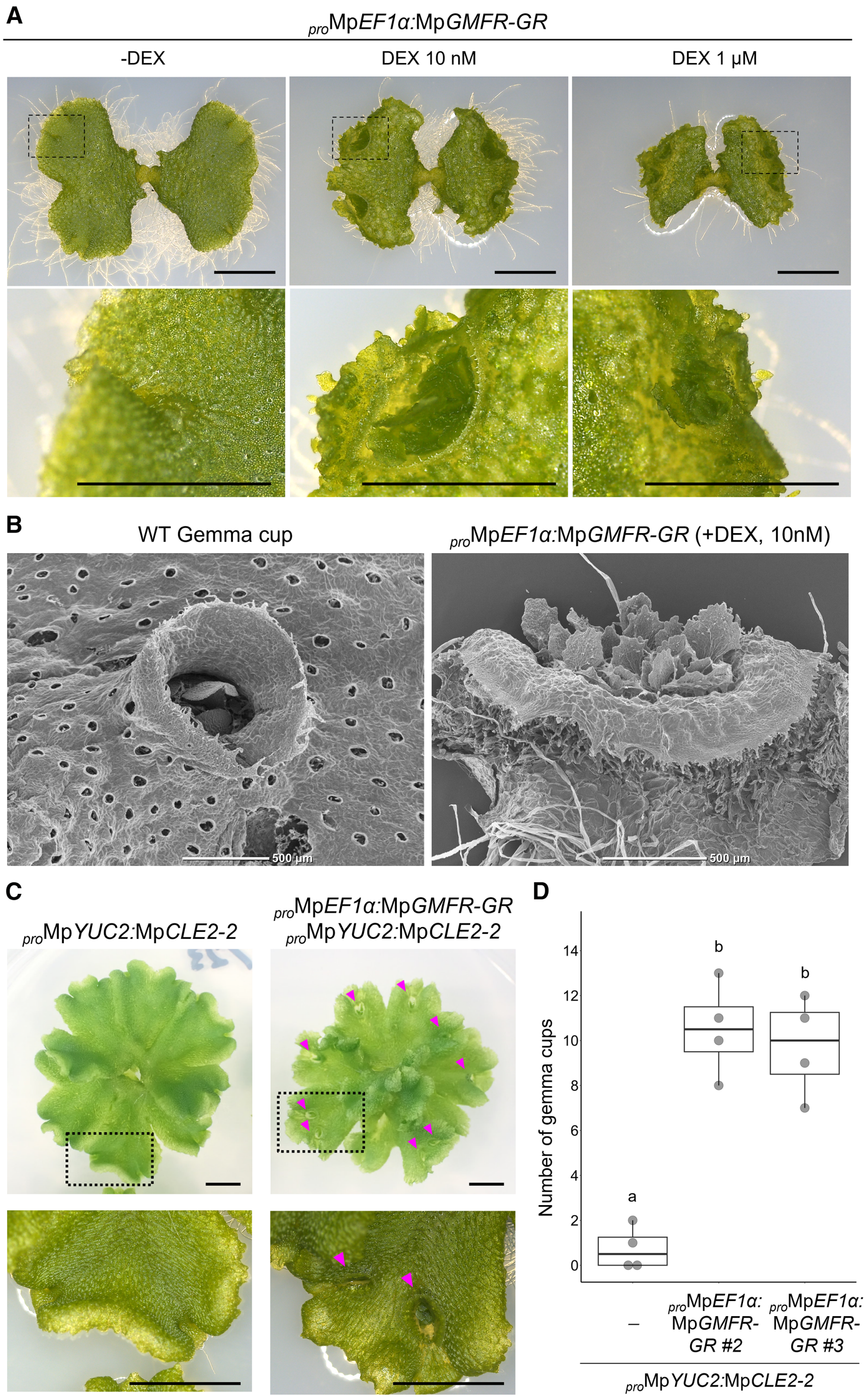

Figure 5. Weak induction of MpGMFR promotes gemma cup formation

(A) Effects of different concentrations of DEX on gemma cup formation in $_{pro}$Mp*EF1α*:Mp*GMFR-GR* plants. 4-day-old gemmalings were transferred from normal medium to DEX-containing medium (0, 10 nM, or 1 μM) and cultured for 7 days. Panels in the bottom row show magnifications of the dashed boxes in the upper panels. Scale bars, 5 mm.

(B) SEM images of the gemma cups observed in 20-day-old wild-type plant and 7-day-old $_{pro}$Mp*EF1α*:Mp*GMFR-GR* plant. Scale bars, 500 μm.

(C) Effects of MpGMFR-GR induction in Mp*CLE2* overexpression plant. 12-day-old plants were transferred from normal medium to 10 nM DEX-containing medium and cultured for 8 days. Panels in the bottom row show magnifications of the dashed boxes in the upper panels. Scale bars, 5 mm.

(D) Quantification of the number of gemma cups shown in (C). The boxes show the median and IQR, and the whiskers extend to 1.5x the IQR. Individual data points are plotted as dots ($n = 4$). Statistical significance was determined by two-way ANOVA with Tukey's post-hoc test; means sharing the same superscript letters are not significantly different, $p < 0.05$.

See also Figure S4.

could not observe either the protruding cells or early-stage gemmae in this line, suggesting that both Mp*GMFR* and Mp*GCAM1* are essential for the initiation of gemma development. Collectively, these data suggest that gemma development is initiated through the stepwise function of Mp*GMFR* and Mp*GCAM1*.

Next, we introduced a frameshift mutation in Mp*GMFR* by using CRISPR-Cas9 genome editing (Mp*gmfr-3^{ge}*) in the $_{pro}$Mp*EF1α*:Mp*GCAM1-GR* background (Figures S1A and S1B). Explants containing an apical notch were grown on mock or 10 μM DEX-containing medium for 10 days. In the background of the Mp*GCAM1-GR* overexpression line, all observed plants formed gemmae and gemma cups under both mock and DEX treatment. In Mp*gmfr-3^{ge}* $_{pro}$Mp*EF1α*:Mp*GCAM1-GR* plants, all observed plants failed to produce gemmae and gemma cups in mock treatment. In contrast, a small proportion of plants (3/18) formed at least 1 gemma cup under DEX treatment (Figure 6F). The induced gemma cups contained detachable gemmae. Compared with normal gemmae, they are malformed and possess 2 or fewer apical notches and a trace of stalk (Figure S6C). The induced gemmae can grow on medium and develop a thallus that lacks gemma cups (Figure S6D). These data show that loss of gemmae and gemma cups in Mp*gmfr* mutants can be partially recovered by overexpression of Mp*GCAM1*, suggesting that Mp*GCAM1* is a key factor that acts downstream of Mp*GMFR*. In addition to the gemma and gemma cup development, Mp*GCAM1* was reported to induce an undifferentiated cell clump when overexpressed.[6] The formation of a cell clump was observed in both $_{pro}$Mp*EF1α*:Mp*GCAM1-GR* and Mp*gmfr-3^{ge}* $_{pro}$Mp*EF1α*:Mp*GCAM1-GR* plants grown on 10 μM DEX-containing medium for 14 days (Figure S6E), suggesting that Mp*GMFR* does not act downstream of Mp*GCAM1* during the cell clump formation.

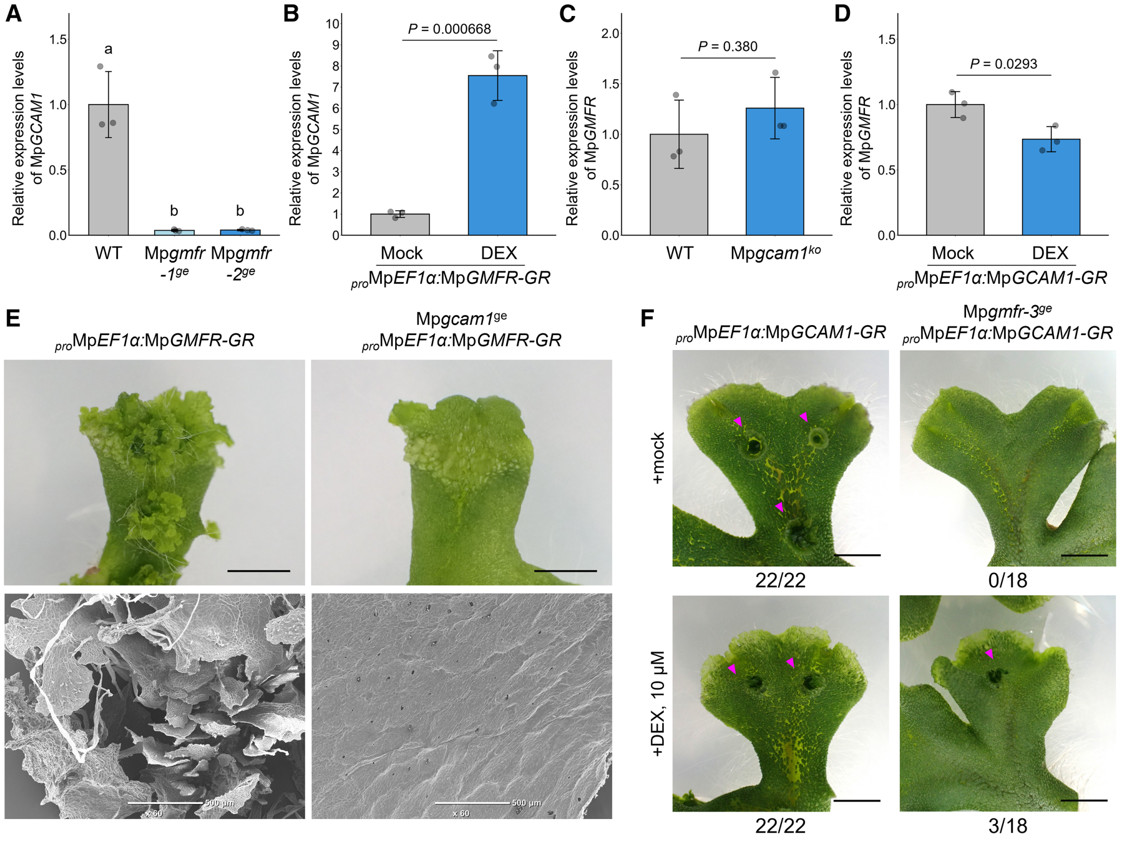

Figure 6. Mp*GCAM1* acts downstream of Mp*GMFR*

(A and B) Relative expression levels of Mp*GMFR*. 6-day-old plants grown from explants (A) and 4-day-old gemmalings (B) are examined.

(C and D) Relative expression levels of Mp*GCAM1*. 6-day-old plants grown from explants (C) and 4-day-old gemmalings grown with mock or 1 μM DEX (D) are examined.

(E) Effects of MpGMFR-GR induction in Mp*gcam1^{ge}* mutant background. Plants were grown on mock or 1 μM DEX-containing medium for 7 days, then transferred to DEX-free medium for an additional 7-days to observe the gemma-inducing activity of Mp*GMFR*. Overall plant morphology near the apical notch (top) and the SEM images of the dorsal surface (bottom) are indicated. Scale bars, 0.5 mm.

(F) Effects of MpGCAM1-GR induction in Mp*gmfr-3^{ge}*. Explants from the tips of thalli were grown with mock or 10 μM DEX for 10 days. Arrowheads indicate gemma cups. The frequency of plants that formed at least 1 gemma cup is indicated below each panel (*n* = 18–22). Scale bars, 0.5 mm.

In (A)–(D), the expression levels were normalized to Mp*APT* and are represented as mean and SD (bars) with individual data points (dots) (*n* = 3). Statistical significance was determined by two-way ANOVA with Tukey's post-hoc test in (A) and Student's *t* test in (B)–(D). Means sharing the same superscript letters are not significantly different, $p < 0.05$ in (A). In (B)–(D), *p* value is indicated above each pair of bars.

See also Figures S1, S5, and S6.

DISCUSSION

Due to its nature of autonomous asexual reproduction, the liverwort *Marchantia polymorpha* has become an important model organism for studying the molecular mechanisms of asexual reproduction. In this study, we identified Mp*ERF14/GMFR* as a key regulator of the initiation of asexual reproduction. Molecular genetic analysis shows that Mp*GMFR* is required for the development of gemma cups and gemmae, while overexpression of Mp*GMFR* induces gemma cups and/or gemmae in a dose-dependent manner. When weakly induced, MpGMFR acts as a positive regulator of formation of gemma cups containing gemmae, which is consistent with the inhibitory function of MpCLE2 in gemma cup development, as the expression of Mp*GMFR* is suppressed by MpCLE2 peptide signaling. Transient and strong induction of MpGMFR results in the formation of ectopic gemmae with a junction to the stalk and meristematic notches, which enables gemmae to be detached from the parental plant and grow independently in a remote location. In contrast to weak induction, which induces the formation of gemma cups and gemmae, transient and strong induction promotes ectopic gemma formation without concomitant gemma cup development. We speculate that this is due to the inhibition of gemma cup formation by the high-level activation of Mp*GMFR*. Furthermore, continuous and strong induction results in defective gemma morphology. Given that the expression of Mp*GMFR*, which begins in the early stage, is progressively restricted to the vicinity of the meristems during gemma development, it is likely that the precise spatiotemporal control of Mp*GMFR* expression is crucial to this process.

Previous studies show that Mp*GCAM1* is required for gemma and gemma cup formation.[6,8–10] In this study, we conducted a

CellPress
OPEN ACCESS

series of molecular genetic analyses to compare the expression and function of Mp*GMFR* and Mp*GCAM1*, revealing that Mp*GMFR* expression precedes Mp*GCAM1* expression during early gemma development. Consistent with this pattern, functional analysis showed that Mp*GCAM1* expression is substantially reduced in Mp*gmfr* mutants, in which gemmae fail to form. Importantly, induction of MpGCAM1-GR in Mp*gmfr* can partially rescue gemma and gemma cup development. These findings suggest that Mp*GMFR* acts as a master regulator that initiates gemma and gemma cup development by activating Mp*GCAM1* as a key downstream effector.

Our phylogenetic analysis suggests that class VIII of AP2/ERF family proteins can be classified into 3 subgroups, each comprising genes from major lineages of land plants, including a single *M. polymorpha* gene (Mp*ERF1*, Mp*ERF20/LAXR,* and Mp*ERF14/GMFR*). Thus, three distinct ERF-VIII genes were already present in the last common ancestor of land plants, whereas their closest homolog is unclear in the ERF family of streptophyte algae.[25] In *Arabidopsis thaliana*, At*ERF84,* which belongs to the subgroup that contains Mp*GMFR,* has been reported as a positive regulator in drought resistance, while its function in development is unclear.[31] In *M. polymorpha*, Mp*ERF20/LAXR* regulates cellular reprogramming during tissue regeneration in response to reduced auxin levels after the decapitation of meristematic notch.[32] Mp*ERF20/LAXR* is a homolog of the *A. thaliana ENHANCER OF SHOOT REGENERATION 1/ DORNRÖSCHEN* (*ESR1/DRN*), which promotes stem cell formation during shoot regeneration.[33] In the moss *P. patens*, Pp*ESR* genes promote gametophore apical cell identity, whose expression is regulated by cytokinin signaling,[34] implying deep evolutionary conservation of these genes in controlling stem cell identity in land plants. The role of Mp*ERF14/GMFR* in asexual reproduction parallels that of the *ESR/LAXR* genes in the regulation of cell identity, as both lead to the *de novo* formation of meristems, even though Mp*ERF20/LAXR* cannot replace the activity of Mp*ERF14/GMFR*.

In summary, we have presented the identification of Mp*ERF14/GMFR* as a molecular trigger for asexual reproduction in *M. polymorpha*. This work provides a foundation for future studies to elucidate how plants evolved the formation of extra meristems from their bodies, which may have made a significant contribution to their prosperity on land.

RESOURCE AVAILABILITY

Lead contact

Further information and requests for resources and reagents should be directed to and will be fulfilled by the lead contact, Yuki Hirakawa (yuki-hirakawa@hiroshima-u.ac.jp).

Materials availability

Please note that the transfer of transgenic plants will be subject to MTA and any relevant import permits.

Data and code availability

- All original microscopic imaging data reported in this study are available from the lead contact upon request.
- This study does not report original code.
- Any additional information required to reanalyze the data reported in this study is available from the lead contact upon request.

ACKNOWLEDGMENTS

We thank Ikuko Nakanomyo, Jutarou Fukazawa, Sayaka Matsui, and Yuuki Sakai for technical assistance. This work was conducted with the facilities at the Natural Science Center for Basic Research and Development (N-BARD) at Hiroshima University (NBARD-00283), which are supported by the MEXT Program for supporting construction of core facilities (grant number JPMXS0441300025). This work was supported by JSPS KAKENHI (grant number JP22H02676) to Y.H.; the Takeda Science Foundation, the Foundation of Kinoshita Memorial Enterprise, the Naito Foundation, and the Sumitomo Foundation to Y.H.; GteX Program Japan (JPMJGX23B0) to K.I.; the Program for Forming Japan's Peak Research Universities (J-PEAKS) from JSPS to K.I.; BBSRC BB/T007117/1 to J.H.; and BBSRC BB/F011458/1 for confocal microscopy. F.R. is a Leverhulme Early Career Fellow (ECF-2023-534) funded by the Leverhulme Trust and the Isaac Newton Trust (23.08(f)), and I.B. is funded by the Herschel Smith Fund studentship.

AUTHOR CONTRIBUTIONS

G.T. and Y.H. conceived and designed the research. G.T., S.Y., F.R., I.B., and M.S. performed the experiments. G.T., S.Y., F.R., M.S., and Y.H. analyzed the data. K.I. contributed materials and analysis tools. F.R., T.K., J.H., and Y.H. supervised the project. G.T. and Y.H. wrote the manuscript. All authors reviewed and edited the manuscript.

DECLARATION OF INTERESTS

The authors declare no competing interests.

STAR★METHODS

Detailed methods are provided in the online version of this paper and include the following:

- KEY RESOURCES TABLE
- EXPERIMENTAL MODEL AND STUDY PARTICIPANT DETAILS
  - Plant materials and growth conditions
- METHOD DETAILS
  - Plasmid construction
  - Generation of transgenic plants
  - Imaging and phenotypic measurement
  - GUS staining
  - RT-qPCR
  - Phylogenetic analysis
  - Data visualization
- QUANTIFICATION AND STATISTICAL ANALYSIS

SUPPLEMENTAL INFORMATION

Supplemental information can be found online at https://doi.org/10.1016/j.cub.2026.03.083.

Received: October 20, 2025
Revised: November 6, 2025
Accepted: March 30, 2026
Published: May 4, 2026

REFERENCES

1. Niklas, K.J., and Cobb, E.D. (2017). The evolutionary ecology (evo-eco) of plant asexual reproduction. Evol. Ecol. *31*, 317–332. https://doi.org/10.1007/s10682-016-9828-5.
2. Imura, S. (1994). Vegetative Diaspores in Japanese Mosses. J. Hattori Bot. Lab. *77*, 177–232. https://doi.org/10.18968/jhbl.77.0_177.
3. Laaka-Lindberg, S., Korpelainen, H., and Pohjamo, M. (2003). Dispersal of Asexual Propagules in Bryophytes. J. Hattori Bot. Lab. *93*, 319–330. https://doi.org/10.18968/jhbl.93.0_319.

4. Shimamura, M. (2016). *Marchantia polymorpha* : Taxonomy, Phylogeny and Morphology of a Model System. Plant Cell Physiol. *57*, 230–256. https://doi.org/10.1093/pcp/pcv192.

5. Kato, H., Yasui, Y., and Ishizaki, K. (2020). Gemma cup and gemma development in *Marchantia polymorpha*. New Phytol. *228*, 459–465. https://doi.org/10.1111/nph.16655.

6. Yasui, Y., Tsukamoto, S., Sugaya, T., Nishihama, R., Wang, Q., Kato, H., Yamato, K.T., Fukaki, H., Mimura, T., Kubo, H., et al. (2019). GEMMA CUP-ASSOCIATED MYB1, an Ortholog of Axillary Meristem Regulators, Is Essential in Vegetative Reproduction in *Marchantia polymorpha*. Curr. Biol. *29*, 3987–3995. https://doi.org/10.1016/j.cub.2019.10.004.

7. Aki, S.S., Mikami, T., Naramoto, S., Nishihama, R., Ishizaki, K., Kojima, M., Takebayashi, Y., Sakakibara, H., Kyozuka, J., Kohchi, T., et al. (2019). Cytokinin Signaling Is Essential for Organ Formation in *Marchantia polymorpha*. Plant Cell Physiol. *60*, 1842–1854. https://doi.org/10.1093/pcp/pcz100.

8. Aki, S.S., Morimoto, T., Ohnishi, T., Oda, A., Kato, H., Ishizaki, K., Nishihama, R., Kohchi, T., and Umeda, M. (2022). R2R3-MYB transcription factor GEMMA CUP-ASSOCIATED MYB1 mediates the cytokinin signal to achieve proper organ development in *Marchantia polymorpha*. Sci. Rep. *12*, 21123. https://doi.org/10.1038/s41598-022-25684-3.

9. Komatsu, A., Kodama, K., Mizuno, Y., Fujibayashi, M., Naramoto, S., and Kyozuka, J. (2023). Control of vegetative reproduction in *Marchantia polymorpha* by the KAI2-ligand signaling pathway. Curr. Biol. *33*, 1196–1210. https://doi.org/10.1016/j.cub.2023.02.022.

10. Komatsu, A., Fujibayashi, M., Kumagai, K., Suzuki, H., Hata, Y., Takebayashi, Y., Kojima, M., Sakakibara, H., and Kyozuka, J. (2025). KAI2-dependent signaling controls vegetative reproduction in *Marchantia polymorpha* through activation of LOG-mediated cytokinin synthesis. Nat. Commun. *16*, 1263. https://doi.org/10.1038/s41467-024-55728-3.

11. Sakai, Y., Takami, H., Yamaoka, S., Kato, H., Fukaki, H., Kohchi, T., and Ishizaki, K. (2025). SHOT GLASS, an R2R3-MYB transcription factor, promotes gemma cup and gametangiophore development in *Marchantia polymorpha*. New Phytol. *247*, 2678–2696. https://doi.org/10.1111/nph.70337.

12. Proust, H., Honkanen, S., Jones, V.A.S., Morieri, G., Prescott, H., Kelly, S., Ishizaki, K., Kohchi, T., and Dolan, L. (2016). *RSL* Class I Genes Controlled the Development of Epidermal Structures in the Common Ancestor of Land Plants. Curr. Biol. *26*, 93–99. https://doi.org/10.1016/j.cub.2015.11.042.

13. Honkanen, S., Thamm, A., Arteaga-Vazquez, M.A., and Dolan, L. (2018). Negative regulation of conserved *RSL* class I bHLH transcription factors evolved independently among land plants. eLife *7*, e38529. https://doi.org/10.7554/eLife.38529.

14. Hiwatashi, T., Goh, H., Yasui, Y., Koh, L.Q., Takami, H., Kajikawa, M., Kirita, H., Kanazawa, T., Minamino, N., Togawa, T., et al. (2019). The RopGEF KARAPPO Is Essential for the Initiation of Vegetative Reproduction in *Marchantia polymorpha*. Curr. Biol. *29*, 3525–3531. https://doi.org/10.1016/j.cub.2019.08.071.

15. Rong, D., Zhao, S., Tang, W., Luo, N., He, H., Wang, Z., Ma, H., Huang, Y., Yao, X., Pan, X., et al. (2022). ROP signaling regulates spatial pattern of cell division and specification of meristem notch. Proc. Natl. Acad. Sci. USA *119*, e2117803119. https://doi.org/10.1073/pnas.2117803119.

16. Mulvey, H., and Dolan, L. (2023). RHO GTPase of plants regulates polarized cell growth and cell division orientation during morphogenesis. Curr. Biol. *33*, 2897–2911. https://doi.org/10.1016/j.cub.2023.06.015.

17. Sakai, Y., Ueno, A., Yonetsuka, H., Goh, T., Kato, H., Kondo, Y., Fukaki, H., and Ishizaki, K. (2024). Regulation of ROP GTPase cycling between active and inactive states is essential for vegetative organogenesis in *Marchantia polymorpha*. Development *151*, dev202928. https://doi.org/10.1242/dev.202928.

18. Kato, H., Kouno, M., Takeda, M., Suzuki, H., Ishizaki, K., Nishihama, R., and Kohchi, T. (2017). The Roles of the Sole Activator-Type Auxin Response Factor in Pattern Formation of *Marchantia polymorpha*. Plant Cell Physiol. *58*, 1642–1651. https://doi.org/10.1093/pcp/pcx095.

19. Monte, I., Franco-Zorrilla, J.M., García-Casado, G., Zamarreño, A.M., García-Mina, J.M., Nishihama, R., Kohchi, T., and Solano, R. (2019). A Single JAZ Repressor Controls the Jasmonate Pathway in *Marchantia polymorpha*. Mol. Plant *12*, 185–198. https://doi.org/10.1016/j.molp.2018.12.017.

20. Li, D., Flores-Sandoval, E., Ahtesham, U., Coleman, A., Clay, J.M., Bowman, J.L., and Chang, C. (2020). Ethylene-independent functions of the ethylene precursor ACC in *Marchantia polymorpha*. Nat. Plants *6*, 1335–1344. https://doi.org/10.1038/s41477-020-00784-y.

21. Hirakawa, Y., Fujimoto, T., Ishida, S., Uchida, N., Sawa, S., Kiyosue, T., Ishizaki, K., Nishihama, R., Kohchi, T., and Bowman, J.L. (2020). Induction of Multichotomous Branching by CLAVATA Peptide in *Marchantia polymorpha*. Curr. Biol. *30*, 3833–3840. https://doi.org/10.1016/j.cub.2020.07.016.

22. Takahashi, G., Betsuyaku, S., Okuzumi, N., Kiyosue, T., and Hirakawa, Y. (2021). An Evolutionarily Conserved Coreceptor Gene Is Essential for CLAVATA Signaling in *Marchantia polymorpha*. Front. Plant Sci. *12*, 657548. https://doi.org/10.3389/fpls.2021.657548.

23. Hirakawa, Y. (2022). Evolution of meristem zonation by CLE gene duplication in land plants. Nat. Plants *8*, 735–740. https://doi.org/10.1038/s41477-022-01199-7.

24. Takahashi, G., Kiyosue, T., and Hirakawa, Y. (2023). Control of stem cell behavior by CLE-JINGASA signaling in the shoot apical meristem in *Marchantia polymorpha*. Curr. Biol. *33*, 5121–5131. https://doi.org/10.1016/j.cub.2023.10.054.

25. Bowman, J.L., Kohchi, T., Yamato, K.T., Jenkins, J., Shu, S., Ishizaki, K., Yamaoka, S., Nishihama, R., Nakamura, Y., Berger, F., et al. (2017). Insights into Land Plant Evolution Garnered from the *Marchantia polymorpha* Genome. Cell *171*, 287–304. https://doi.org/10.1016/j.cell.2017.09.030.

26. Montgomery, S.A., Tanizawa, Y., Galik, B., Wang, N., Ito, T., Mochizuki, T., Akimcheva, S., Bowman, J.L., Cognat, V., Maréchal-Drouard, L., et al. (2020). Chromatin Organization in Early Land Plants Reveals an Ancestral Association between H3K27me3, Transposons, and Constitutive Heterochromatin. Curr. Biol. *30*, 573–588.e7. https://doi.org/10.1016/j.cub.2019.12.015.

27. Mirbel, C.-F. (1835). Researches anatomiques et physiologiques sur le Marchantia polymorpha. Mém, Acad. R. Sci. Inst. Fr. *13*, 337–436.

28. Barnes, C.R., and Land, W.J.G. (1908). Bryological Papers. II. The Origin of the Cupule of Marchantia. Botanical Gazette *46*, 401–409. https://doi.org/10.1086/329782.

29. Sakamoto, Y., Ishimoto, A., Sakai, Y., Sato, M., Nishihama, R., Abe, K., Sano, Y., Furuichi, T., Tsuji, H., Kohchi, T., et al. (2022). Improved clearing method contributes to deep imaging of plant organs. Commun. Biol. *5*, 12. https://doi.org/10.1038/s42003-021-02955-9.

30. Romani, F., Sauret-Güeto, S., Rebmann, M., Annese, D., Bonter, I., Tomaselli, M., Dierschke, T., Delmans, M., Frangedakis, E., Silvestri, L., et al. (2024). The landscape of transcription factor promoter activity during vegetative development in Marchantia. Plant Cell *36*, 2140–2159. https://doi.org/10.1093/plcell/koae053.

31. Zhang, P., He, R., Yang, J., Cai, J., Qu, Z., Yang, R., Gu, J., Wang, Z.Y., Adelson, D.L., Zhu, Y., et al. (2023). The long non-coding RNA *DANA2* positively regulates drought tolerance by recruiting ERF84 to promote JMJ29-mediated histone demethylation. Mol. Plant *16*, 1339–1353. https://doi.org/10.1016/j.molp.2023.08.001.

32. Ishida, S., Suzuki, H., Iwaki, A., Kawamura, S., Yamaoka, S., Kojima, M., Takebayashi, Y., Yamaguchi, K., Shigenobu, S., Sakakibara, H., et al. (2022). Diminished Auxin Signaling Triggers Cellular Reprogramming by Inducing a Regeneration Factor in the Liverwort *Marchantia polymorpha*. Plant Cell Physiol. *63*, 384–400. https://doi.org/10.1093/pcp/pcac004.

33. Banno, H., Ikeda, Y., Niu, Q.W., and Chua, N.H. (2001). Overexpression of Arabidopsis *ESR1* Induces Initiation of Shoot Regeneration. Plant Cell *13*, 2609–2618. https://doi.org/10.1105/tpc.010234.

34. Hata, Y., Hetherington, N., Battenberg, K., Hirota, A., Minoda, A., Hayashi, M., and Kyozuka, J. (2025). snRNA-seq analysis of the moss *Physcomitrium patens* identifies a conserved cytokinin-ESR module promoting pluripotent stem cell identity. Dev. Cell *60*, 1884–1899. https://doi.org/10.1016/j.devcel.2025.02.006.

35. Tanizawa, Y., Mochizuki, T., Yagura, M., Sakamoto, M., Fujisawa, T., Kawamura, S., Shimokawa, E., Yamaoka, S., Nishihama, R., Bowman, J.L., and Berger, F. (2025). MarpolBase: Genome Database for *Marchantia polymorpha* Featuring High Quality Reference Genome Sequences. Plant. Cell Physiol. pcaf159.

36. Ishizaki, K., Nishihama, R., Yamato, K.T., and Kohchi, T. (2016). Molecular Genetic Tools and Techniques for *Marchantia polymorpha* Research. Plant Cell Physiol. *57*, 262–270. https://doi.org/10.1093/pcp/pcv097.

37. Ishizaki, K., Nishihama, R., Ueda, M., Inoue, K., Ishida, S., Nishimura, Y., Shikanai, T., and Kohchi, T. (2015). Development of Gateway Binary Vector Series with Four Different Selection Markers for the Liverwort *Marchantia polymorpha*. PLoS One *10*, e0138876. https://doi.org/10.1371/journal.pone.0138876.

38. Sugano, S.S., Nishihama, R., Shirakawa, M., Takagi, J., Matsuda, Y., Ishida, S., Shimada, T., Hara-Nishimura, I., Osakabe, K., and Kohchi, T. (2018). Efficient CRISPR/Cas9-based genome editing and its application to conditional genetic analysis in *Marchantia polymorpha*. PLoS One *13*, e0205117. https://doi.org/10.1371/journal.pone.0205117.

39. Naito, Y., Hino, K., Bono, H., and Ui-Tei, K. (2015). CRISPRdirect: software for designing CRISPR/Cas guide RNA with reduced off-target sites. Bioinformatics *31*, 1120–1123. https://doi.org/10.1093/bioinformatics/btu743.

40. Schindelin, J., Arganda-Carreras, I., Frise, E., Kaynig, V., Longair, M., Pietzsch, T., Preibisch, S., Rueden, C., Saalfeld, S., Schmid, B., et al. (2012). Fiji: an open-source platform for biological-image analysis. Nat. Methods *9*, 676–682. https://doi.org/10.1038/nmeth.2019.

41. Gouy, M., Tannier, E., Comte, N., and Parsons, D.P. (2021). Seaview version 5: A multiplatform software for multiple sequence alignment, molecular phylogenetic analyses, and tree reconciliation. Methods Mol. Biol. *2231*, 241–260. https://doi.org/10.1007/978-1-0716-1036-7_15.

42. Ronquist, F., Teslenko, M., van der Mark, P., Ayres, D.L., Darling, A., Höhna, S., Larget, B., Liu, L., Suchard, M.A., and Huelsenbeck, J.P. (2012). MrBayes 3.2: Efficient Bayesian Phylogenetic Inference and Model Choice Across a Large Model Space. Syst. Biol. *61*, 539–542. https://doi.org/10.1093/sysbio/sys029.

43. Flores-Sandoval, E., Dierschke, T., Fisher, T.J., and Bowman, J.L. (2016). Efficient and Inducible Use of Artificial MicroRNAs in *Marchantia polymorpha*. Plant Cell Physiol. *57*, 281–290. https://doi.org/10.1093/pcp/pcv068.

44. Romani, F., Bonter, I., Rebmann, M., Takahashi, G., Guzman-Chavez, F., De Batté, F., Hirakawa, Y., and Haseloff, J. (2026). A simple cell-cycle control system in *Marchantia polymorpha* provides a framework for understanding plant cell proliferation. Plant *koag103*. https://doi.org/10.1093/plcell/koag103.

45. Kubota, A., Ishizaki, K., Hosaka, M., and Kohchi, T. (2013). Efficient Agrobacterium-Mediated Transformation of the Liverwort *Marchantia polymorpha* Using Regenerating Thalli. Biosci. Biotechnol. Biochem. *77*, 167–172. https://doi.org/10.1271/bbb.120700.

46. Goodstein, D.M., Shu, S., Howson, R., Neupane, R., Hayes, R.D., Fazo, J., Mitros, T., Dirks, W., Hellsten, U., Putnam, N., et al. (2012). Phytozome: a comparative platform for green plant genomics. Nucleic Acids Res. *40*, D1178–D1186. https://doi.org/10.1093/nar/gkr944.

47. Gu, K.J., Lin, C.F., Wu, J.J., and Zhao, Y.P. (2022). GinkgoDB: an ecological genome database for the living fossil, Ginkgo biloba. Database (Oxford) *2022*, baac046. https://doi.org/10.1093/database/baac046.

48. Gelman, A., and Rubin, D.B. (1992). Inference from Iterative Simulation Using Multiple Sequences. Statist. Sci. *7*, 457–472. https://doi.org/10.1214/ss/1177011136.

STAR★METHODS

KEY RESOURCES TABLE

REAGENT or RESOURCE	SOURCE	IDENTIFIER
Bacterial and virus strains		
Agrobacterium tumefaciens GV3101 (MP90)	Widely distributed	N/A
Chemicals, peptides, and recombinant proteins		
4% Paraformaldehyde, Phosphate Buffer Solution	Nacalai Tesque	Cat# 09154-14
Tissue-Clearing Reagent iTOMEI-D [for Plants]	Tokyo Chemical Industry	Cat# T3940
Tissue-Clearing Reagent iTOMEI-M (RI 1.40) [for Plants]	Tokyo Chemical Industry	Cat# T4003
SCRI Renaissance 2200	Renaissance Chemicals	N/A
Glutaraldehyde 8% solution	TAAB Laboratories Equipment Ltd.	Cat# G018/1
Osmium Tetroxide 2% w/v solution	TAAB Laboratories Equipment Ltd.	Cat# O018/1
t-Butyl Alcohol	Nacalai Tesque	Cat# 06104-25
Critical commercial assays		
KOD One PCR Master Mix -Blue	Toyobo	Cat# KMM-201
In-Fusion HD Cloning Kit	Takara Bio	Cat# 639633
Gateway LR Clonase II Enzyme mix	Thermo Fisher Scientific	Cat# 11790120
Nucleospin RNA Plant	Macherey-Nagel	Cat# U949B
ReverTra Ace qPCR RT Master Mix with gDNA Remover	Toyobo	Cat# FSQ-301
TB Green Premix Ex Taq II(Tli RNaseH Plus)	Takara Bio	Cat# RR820S
THUNDERBIRD Next SYBR qPCR Mix	Toyobo	Cat# QPX-201
Deposited data		
Marchantia polymorpha MarpolBase	Tanizawa et al.[35]	http://marchantia.info
Arabidopsis thaliana TAIR10	TAIR	https://www.arabidopsis.org/
Ginkgo biloba GinkgoDB	GinkgoDB	https://ginkgo.zju.edu.cn/genome/
Amborella trichopoda v1.0	Amborella Genome Project	https://phytozome.jgi.doe.gov/pz/portal.html
Salvinia cucullata v1.1	MarpolBase	http://marchantia.info; https://fernbase.org/
Selaginella moellendorffii v1.0	Phytozome	https://phytozome.jgi.doe.gov/pz/portal.html
Sphagnum fallax v1.1	Phytozome	https://phytozome.jgi.doe.gov/pz/portal.html
Physcomitrium patens v3.3	Phytozome	https://phytozome.jgi.doe.gov/pz/portal.html
Anthoceros agrestis v1.0	MarpolBase	http://marchantia.info; https://www.hornworts.uzh.ch/en/Blast.html
Experimental models: Organisms/strains		
Marchantia polymorpha Tak-1	Ishizaki et al.[36]	N/A
Marchantia polymorpha Tak-1 proMpYUC2:GUS#2 (pMpGWB204)	Takahashi et al.[24]	N/A
Marchantia polymorpha Tak-1 proMpYUC2:GUS#2 *Mpgmfr-1^{ge}* (pMpGE010)	This paper	N/A
Marchantia polymorpha Tak-1 proMpYUC2:GUS#2 *Mpgmfr-2^{ge}* (pMpGE010)	This paper	N/A
Marchantia polymorpha Tak-1 proMpYUC2:GUS#2 *Mpgmfr-2^{ge}* proMpGMFR:MpGMFR#1 (pMpGWB301)	This paper	N/A
Marchantia polymorpha Tak-1 proMpYUC2:GUS#2 *Mpgmfr-2^{ge}* proMpGMFR:MpGMFR#3 (pMpGWB301)	This paper	N/A
Marchantia polymorpha Tak-1 proMpYUC2:GUS#2 proMpE2F:XVE>>amiR-MpGMFR #2 (pMpGWB368)	This paper	N/A

(*Continued on next page*)

CellPress
OPEN ACCESS

Continued

REAGENT or RESOURCE	SOURCE	IDENTIFIER
Marchantia polymorpha Tak-1 proMpYUC2:GUS#2 *Mpgmfr-2^{ge}* proMpGMFR:MpGMFR-GR#7 (pMpGWB312)	This paper	N/A
Marchantia polymorpha Tak-1 proMpYUC2:GUS#2 *Mpgmfr-2^{ge}* proMpGMFR:MpGMFR-GR#8 (pMpGWB312)	This paper	N/A
Marchantia polymorpha Tak-1 proMpYUC2:GUS#2 *Mpgmfr-2^{ge}* proMpGMFR:MpERF1-GR#1 (pMpGWB312)	This paper	N/A
Marchantia polymorpha Tak-1 proMpYUC2:GUS#2 *Mpgmfr-2^{ge}* proMpGMFR:MpERF1-GR#2 (pMpGWB312)	This paper	N/A
Marchantia polymorpha Tak-1 proMpYUC2:GUS#2 *Mpgmfr-2^{ge}* proMpGMFR:MpLAXR-GR#1 (pMpGWB312)	This paper	N/A
Marchantia polymorpha Tak-1 proMpYUC2:GUS#2 *Mpgmfr-2^{ge}* proMpGMFR:MpLAXR-GR#2 (pMpGWB312)	This paper	N/A
Marchantia polymorpha Tak-1 proMpLAXR:tdTomato-NLS proMpGMFR:H2B-3xCitrine #1 (pMpGWB323)	This paper	N/A
Marchantia polymorpha Tak-1 proMpEF1α:MpGMFR (pMpGWB303)	This paper	N/A
Marchantia polymorpha Tak-1 proMpYUC2:GUS#2 proMpEF1α:MpGMFR-GR #1 (pMpGWB113)	This paper	N/A
Marchantia polymorpha Tak-1 proMpYUC2:GUS#2 proMpEF1α:MpGMFR-GR #2 (pMpGWB113)	This paper	N/A
Marchantia polymorpha Tak-1 proMpEF1α:MpERF1-GR #1 (pMpGWB313)	This paper	N/A
Marchantia polymorpha Tak-1 proMpEF1α:MpLAXR-GR #1 (pMpGWB313)	This paper	N/A
Marchantia polymorpha Tak-1 proMpYUC2: MpCLE2#2 (pMpGWB301)	Hirakawa et al.[21]	N/A
Marchantia polymorpha Tak-1 proMpYUC2:MpCLE2#2 proMpEF1α:MpGMFR-GR #2 (pMpGWB113)	This paper	N/A
Marchantia polymorpha Tak-1 proMpYUC2:MpCLE2#2 proMpEF1α:MpGMFR-GR #3 (pMpGWB113)	This paper	N/A
Marchantia polymorpha MpGCAM1-Citrine knockin	Yasui et al.[6]	N/A
Marchantia polymorpha Tak-1 proMpEF1α:MpGCAM1-GR #1 (pMpGWB313)	Yasui et al.[6]	N/A
Marchantia polymorpha Tak-1 proMpEF1α:MpGCAM1-GR<br>#1 *Mpgmfr-3^{ge}* (pMpGE010)	This paper	N/A
Marchantia polymorpha Tak-1 proMpYUC2:GUS#2 proMpEF1α:MpGMFR-GR #2 *Mpgcam1^{ge}* (pMpGE011)	This paper	N/A
Oligonucleotides		
See Table S1	This paper	N/A
Recombinant DNA		
pMpGWB113	Ishizaki et al.[37]	GenBank: LC057455
pMpGWB204	Ishizaki et al.[37]	GenBank: LC057483
pMpGWB301	Ishizaki et al.[37]	GenBank: LC057517
pMpGWB303	Ishizaki et al.[37]	GenBank: LC057519
pMpGWB312	Ishizaki et al.[37]	GenBank: LC057528
pMpGWB313	Ishizaki et al.[37]	GenBank: LC057529
pMpGWB323	Ishizaki et al.[37]	GenBank: LC057539
pMpGWB368	Ishida et al.[32]	N/A
pMpGE010	Sugano et al.[38]	GenBank: LC090756
pMpGE011	Sugano et al.[38]	GenBank: LC090757

(*Continued on next page*)

Continued

REAGENT or RESOURCE	SOURCE	IDENTIFIER
Software and algorithms		
CRISPRdirect	Naito et al.[39]	RRID: SCR_018186
Fiji	Schindelin et al.[40]	RRID: SCR_002285
CLUSTALW	DDBJ	RRID: SCR_017277
SeaView	Gouy et al.[41]	RRID: SCR_015059
MrBayes3.2.7	Ronquist et al.[42]	RRID: SCR_012067
R version 4.5.0 (2025-04-11)	R Core Team, Vienna, Austria	https://www.R-project.org
Rstudio version 2024.12.1 Build 563	RStudio Team; Boston, USA	https://rstudio.com
JMP Pro 18	SAS Institute Inc., North Carolina, USA	https://www.jmp.com/ja/software/predictive-analytics-software

EXPERIMENTAL MODEL AND STUDY PARTICIPANT DETAILS

Plant materials and growth conditions

Marchantia polymorpha male Takaragaike-1 (Tak-1) accession[36] was used as wild type in this study. *M. polymorpha* plants were grown on half-strength Gamborg B5 medium (pH 5.5) solidified with 1.4% agar. *M. polymorpha* plants were grown at 22 °C under continuous white light. Transgenic plants are listed in the key resources table.

METHOD DETAILS

Plasmid construction

Primers used in this study are listed in Table S1. The Gateway cloning system (Thermo Fisher Scientific, Waltham, MA, United States) was used for generation of plant transformation vectors. Gateway destination vectors are described in Ishida et al.[32], Ishizaki et al.[37] and Sugano et al.[38]

For genome editing of Mp*GMFR*/Mp*ERF14* and Mp*GCAM1*, guide RNAs were designed using CRISPRdirect.[39] The plasmids for genome editing were constructed according to Sugano et al.[38]

For the complementation study of a genome editing allele of Mp*GMFR*, a 5026 bp Mp*GMFR* promoter sequence was PCR amplified from *M. polymorpha* genomic DNA with a primer pair of MpGMFR_prom_F_InFusion_XbaI and MpGMFR_prom_R_InFusion_XbaI, and cloned into the Xba I digestion site of pMpGWB301 and pMpGWB312 vectors using In-Fusion HD Cloning Kit (Takara Bio, Shiga, Japan) to produce pMpGWB301-proMpGMFR and pMpGWB312-proMpGMFR, respectively. The coding sequence of Mp*GMFR* was PCR amplified from *M. polymorpha* cDNA with a primer pair of MpGMFR_CDS_F and MpGMFR_CDS_R_+stop or MpGMFR_CDS_R_-stop, and cloned into the Nco I and Xho I digestion sites of pENTR4 Dual Selection Vector (Thermo Fisher Scientific). To introduce gRNA-resistant mutation to the resulting plasmids, pENTR-MpGMFR_CDS_+stop and pENTR-MpGMFR_CDS_-stop, site directed mutagenesis was performed. The plasmids were PCR amplified with mutagenesis primers, MpGMFR_CDS_gRNAres_F and MpGMFR_CDS_gRNAres_R, and subjected to digestion with Dpn I (Takara Bio), followed by transformation of *Escherichia coli*. Mutagenized plasmids were selected by DNA sequencing. pENTR-MpGMFR_CDS_gRNAres_+stop was transferred to pMpGWB301-proMpGMFR, and pENTR-MpGMFR_CDS_gRNAres_-stop was transferred to pMpGWB312-proMpGMFR using Gateway LR Clonase II Enzyme mix (Thermo Fisher Scientific).

For production of the estrogen-inducible artificial microRNA (amiRNA) lines, an amiRNA target sequence was designed at coding sequence of Mp*GMFR* using amiRNA Design Helper[43] (https://marchantia.info/tools/amir_helper/) to be inserted into Mp*MIR160* backbone. Cloning into the pMpGWB368[32] plasmid to generate estrogen-inducible amiRNA construct was performed according to Sakai et al.[17]

For promoter reporter analysis, a 5026 bp DNA fragment of Mp*GMFR* promoter sequence flanking the translational initiation site was PCR amplified with a primer pair of MpGMFR_prom_F and MpGMFR_prom_R, and cloned into pENTR/D-TOPO vector (Thermo Fisher Scientific). The resulting plasmid, pENTR-proMpGMFR, was transferred to the pMpGWB323-H2B vector[24] using Gateway LR Clonase II Enzyme mix.

For production of Mp*GMFR* overexpression alleles, pENTR-MpGMFR_CDS_+stop was transferred to pMpGWB303 using Gateway LR Clonase II Enzyme mix. For production of inducible Mp*GMFR* overexpression alleles, pENTR-MpGMFR_CDS_-stop was transferred to pMpGWB113. For the $_{pro}$*35S*:Mp*GMFR-mVenus*, the CDS of Mp*GMFR* was synthesized (Genewiz) as an L0_CDS12 part and directly cloned into the pBy12 vector as described in Romani et al.[44]

For the functional analysis for Mp*ERF1* and Mp*ERF20/LAXR*, the coding sequences of Mp*ERF1* and Mp*ERF20/LAXR* were PCR amplified from *M. polymorpha* cDNA with a primer pair of MpERF1_CDS_F and MpERF1_CDS_R_-stop or MpLAXR_CDS_F and

MpLAXR_CDS_R_-stop, and cloned into the Nco I and Xho I digestion sites of pENTR4 Dual Selection Vector. The resulting plasmid, pENTR-MpERF1_CDS_-stop or pENTR-MpLAXR_CDS_-stop were transferred into pMpGWB313 or pMpGWB312-proMpGMFR using Gateway LR Clonase II Enzyme mix.

Generation of transgenic plants

Agrobacterium-mediated transformation of *M. polymorpha* was performed using regenerating thalli according to Kubota et al.[45] CRISPR/Cas9-based genome editing was performed according to Sugano et al.[38] Mutations in the guide RNA target loci were examined by direct sequencing of PCR product amplified from genome DNA samples with primers listed in Table S1.

Imaging and phenotypic measurement

For the analysis of overall plant morphology and the measurement for the number of gemma cups, plants were imaged under a digital microscope (DMS1000, Leica Microsystems, Wetzlar, Germany) or a digital camera (TG-6, Olympus).

For fluorescence observation in confocal imaging, plants were fixed and cleared with iTOMEI protocol[29] as described in Takahashi et al.[24] The cleared samples were mounted in the mounting solution and observed under a confocal laser scanning microscopy (Fluoview FV3000, Olympus). For the observation of Citrine fluorescence in developing gemmae, gemma cups and apical notches, hand-sections were prepared with a scalpel. For 3D-reconstruction, Z-series images were processed using '3D-viewer' or '3D-projection' function of Fiji software.[40]

For scanning electron microscopy imaging, plants were pre-fixed with 4 % glutaraldehyde in 50 mM phosphate buffer (pH 7.2) for 2 hours at room temperature, followed by washing in the phosphate buffer. Pre-fixed plants were post-fixed with 1 % osmium tetroxide in 50 mM phosphate buffer (pH 7.2) for 2 hours at 4 °C. Fixed plants were dehydrated using ethanol series at room temperature, then immersed in *t*-butyl alcohol and freeze-dried in an evacuator (VFD-21S, Vacuum Device Inc.) until completely dry. Finally, samples were coated with gold by an ion sputtering device (JFC1500, JEOL) and observed under a scanning electron microscopy (JSM-T220A, JEOL).

GUS staining

GUS staining was performed according to Hirakawa et al.[21] Briefly, individual plants were stained separately in 30–50 μL GUS staining solution (50 mM sodium phosphate buffer pH 7.2, 1 mM potassium-ferrocyanide, 1 mM potassium-ferricyanide, 10 mM EDTA, 0.01 % Triton X-100 and 1mM 5-bromo-4-chloro-3-indolyl-b-D-glucuronic acid) at 37 °C in dark. GUS-stained samples were washed with water, cleared with ethanol, and mounted with clearing solution (chloral hydrate-glycerol-water, 8:1:2) for imaging under a light microscope (BX51, Olympus, Tokyo, Japan).

RT-qPCR

To quantify Mp*GMFR* and Mp*GCAM1* mRNA levels, total RNA was extracted from thallus using NucleoSpin RNA Plant (Macherey-Nagel, Duren, Germany) according to manufacturer's instruction. First-strand cDNAs were prepared by reverse transcriptase (RT) using ReverTra Ace qPCR RT Master Mix with gDNA Remover (TOYOBO, Osaka, Japan). RT-qPCR was performed with the reagents, TB Green Premix Ex Taq II (Takara Bio) and THUNDERBIRD Next SYBR qPCR Mix (TOYOBO), using the devices, Step One Plus Real-Time PCR System (Thermo Fisher Scientific) and CFX Connect (Bio-Rad Laboratories, California, USA). Mp*APT* (Mp3g25140) was used as a reference gene.

Phylogenetic analysis

Protein sequences were retrieved from the following databases: MarpolBase[35] (https://marchantia.info), Phytozome[46] (https://phytozome-next.jgi.doe.gov/), TAIR (http://www.arabidopsis.org/) and GinkgoDB[47] (https://ginkgo.zju.edu.cn/genome/). Alignment was performed on the amino acid sequences of the AP2 domain using CLUSTALW (https://www.genome.jp/tools-bin/clustalw). After manually removing the alignment gaps using SeaView,[41] phylogenetic analysis was performed on the alignment using MrBayes3.2.7.[42] Two runs with four chains of Markov chain Monte Carlo (MCMC) iterations were performed for 6,000,000 generations, keeping one tree every 100 generations. The first 25% of the generations were discarded as burn-in and the remaining trees were used to calculate a 50% majority-rule tree. The standard deviation for the two MCMC iteration runs was below 0.01, suggesting that it was sufficient for the convergence of the two runs. Convergence was assessed by visual inspection of the plot of the log likelihood scores of the two runs calculated by MrBayes.[48] Character matrix used to run the Bayesian phylogenetic analysis is provided in Data S1.

Data visualization

The statistical software R version 4.5.0 was used for data visualization.

QUANTIFICATION AND STATISTICAL ANALYSIS

For phenotypic quantification, JMP pro 18 (JMP Statistical Discovery LLC, North Carolina, USA) was used for statistical tests. Statistical details including the type of test, sample size and statistical significance can be found in figure legends.

Current Biology, Volume *36*

Supplemental Information

Initiation of asexual reproduction by the AP2/ERF gene *GEMMIFER* in *Marchantia polymorpha*

Go Takahashi, Saori Yamaya, Facundo Romani, Ignacy Bonter, Kimitsune Ishizaki, Masaki Shimamura, Tomohiro Kiyosue, Jim Haseloff, and Yuki Hirakawa

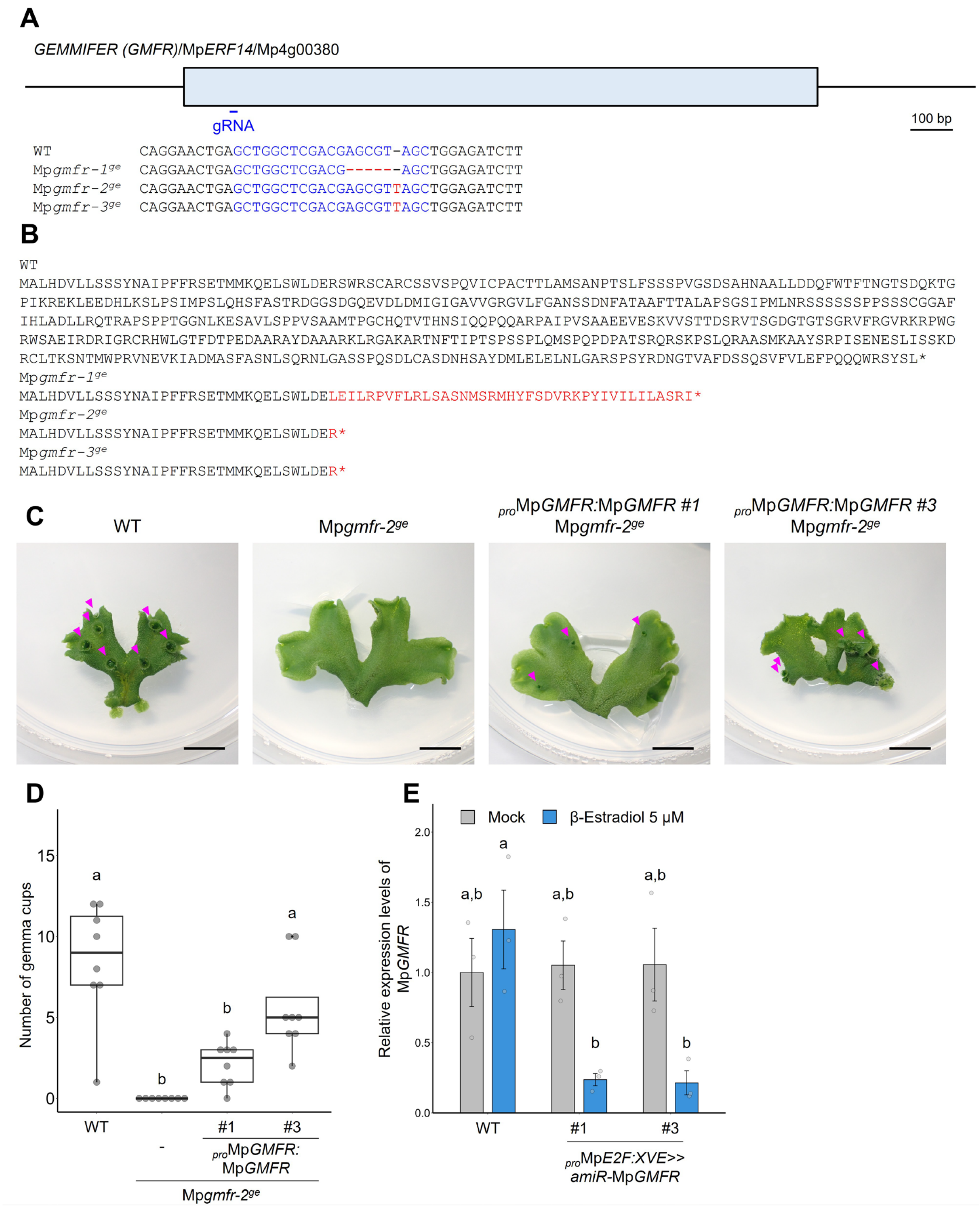

Figure S1. Loss-of-function mutants for *GEMMIFER*. Related to Figures 1 and 6.
(**A**) Structure of Mp*GMFR*/Mp*ERF14*/Mp4g00380 locus with the position of designed guide RNA (gRNA). An exon is shown as a box. Genotyping of genome editing alleles is indicated below. gRNA sequences are in blue. Deleted or inserted bases are in red. (**B**) Deduced MpGMFR protein sequences for WT and mutants. Sequences different from WT are in red. Asterisks indicate translational termination. (**C**) Complementation test for Mp*gmfr-2*^{ge} by gRNA-resistant Mp*GMFR* coding sequence driven under own promoter. Arrowheads indicate gemma cups. Scale bars, 1 cm. (**D**) The number of gemma cups (n=8). (**E**) Relative expression levels of Mp*GMFR* in 14-day-old plants of wild type and $_{pro}$Mp*E2F:XVE>>amiR*-Mp*GMFR* grown from gemmae in mock or β-estradiol-containing medium, normalized by Mp*APT* (n=3, biological replicates). In (**D**)**,** the boxes show the median and interquartile range (IQR), and the whiskers extend to 1.5× IQR. Individual data points are plotted as dots. In (**E**)**,** data are represented by mean and SD (bars) with individual data points (dots). Statistical significance was determined by two-way ANOVA with Tukey's post hoc test in (**D, E**). Means sharing the same superscript letters are not significantly different, $p < 0.05$.

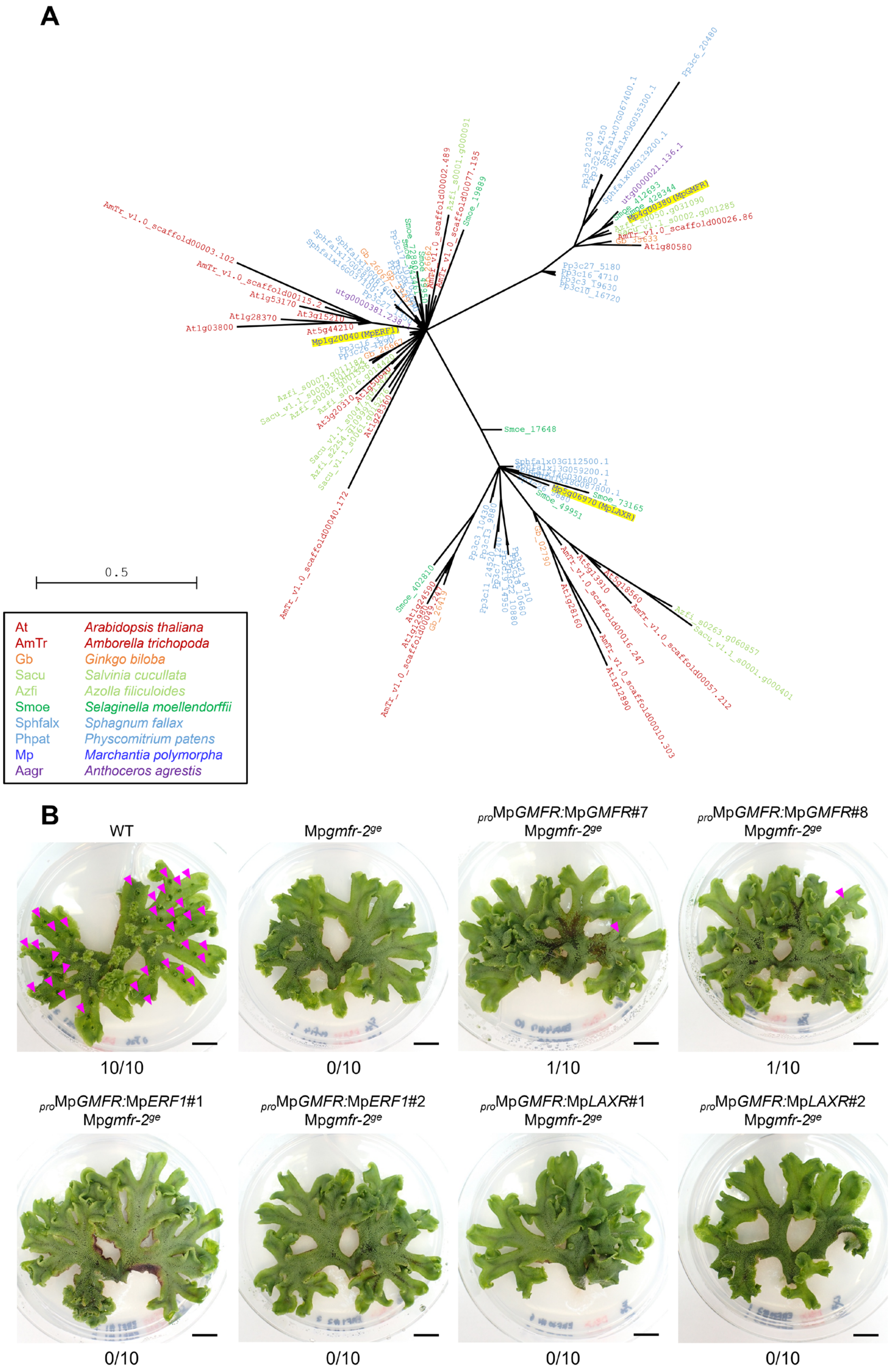

Figure S2. Analysis of ERF-VIII genes. Related to Figure 1.
(**A**) Molecular phylogenetic tree of class VIII of AP2/ERF family among land plant species, generated with a Bayesian method based on the conserved AP2 domain. (**B**) Complementation test of Mp*gmfr*^{ge} by Mp*GMFR-GR*, Mp*ERF1-GR* and Mp*LAXR-GR* driven under Mp*GMFR* promoter. The frequency of plants containing at least one gemma cup is indicated below each panel (n=10). Arrowheads indicate gemma cups. Scale bars, 1 cm.

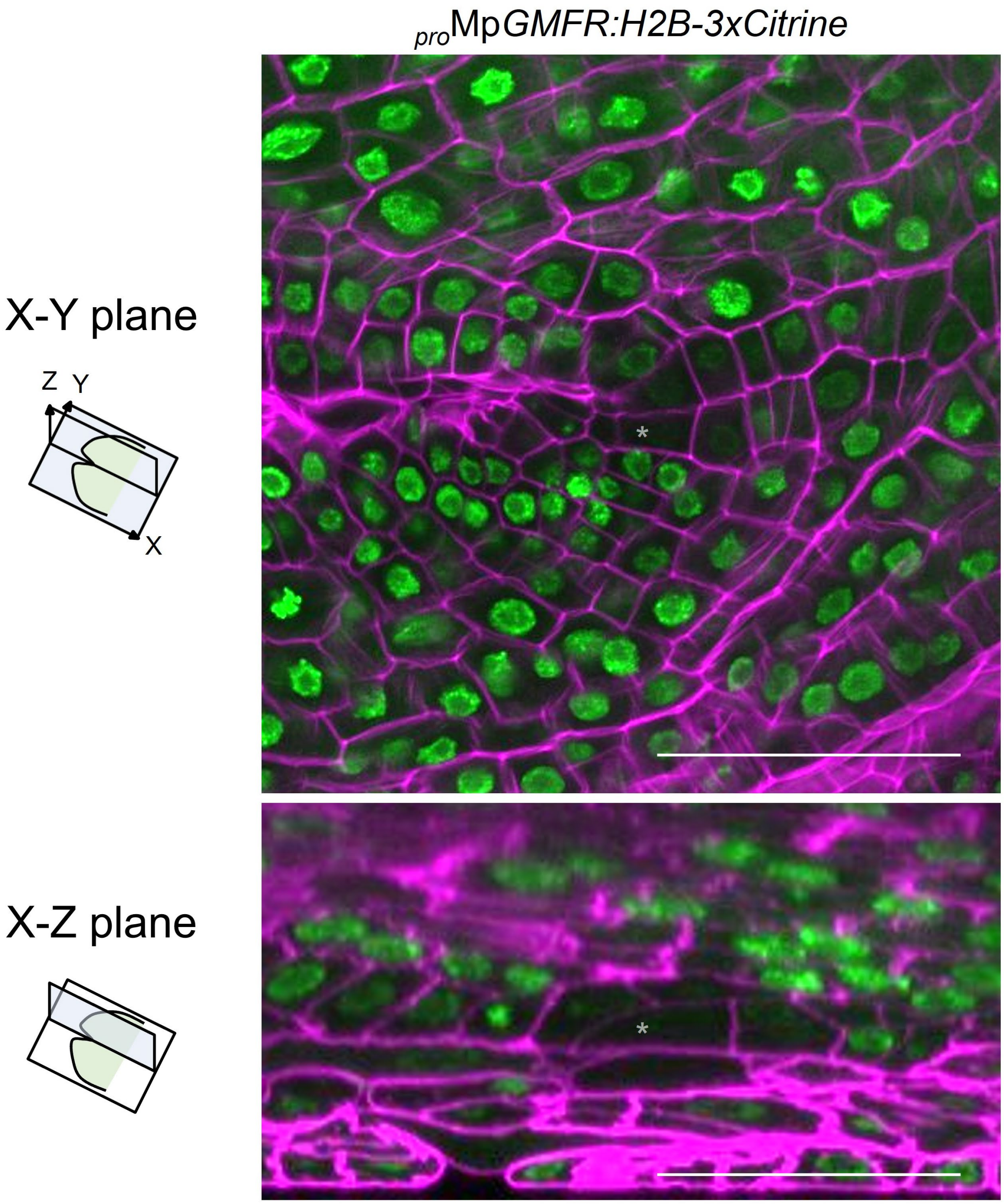

Figure S3. Expression patterns of Mp*GMFR* around the stem cell zone. Related to Figure 2.
Confocal imaging of the apical notch of $_{pro}$Mp*GMFR:H2B-3xCitrine* in 23-day-old plant. A single X-Y plane (top) and a reconstructed X-Z orthogonal view (bottom) derived from Z-stack images are indicated. Cell walls were stained with SCRI Renaissance 2200 (SR2200). Asterisks indicate a (sub)apical cell observed in the X-Y and X-Z planes. Scale bars, 50 µm.

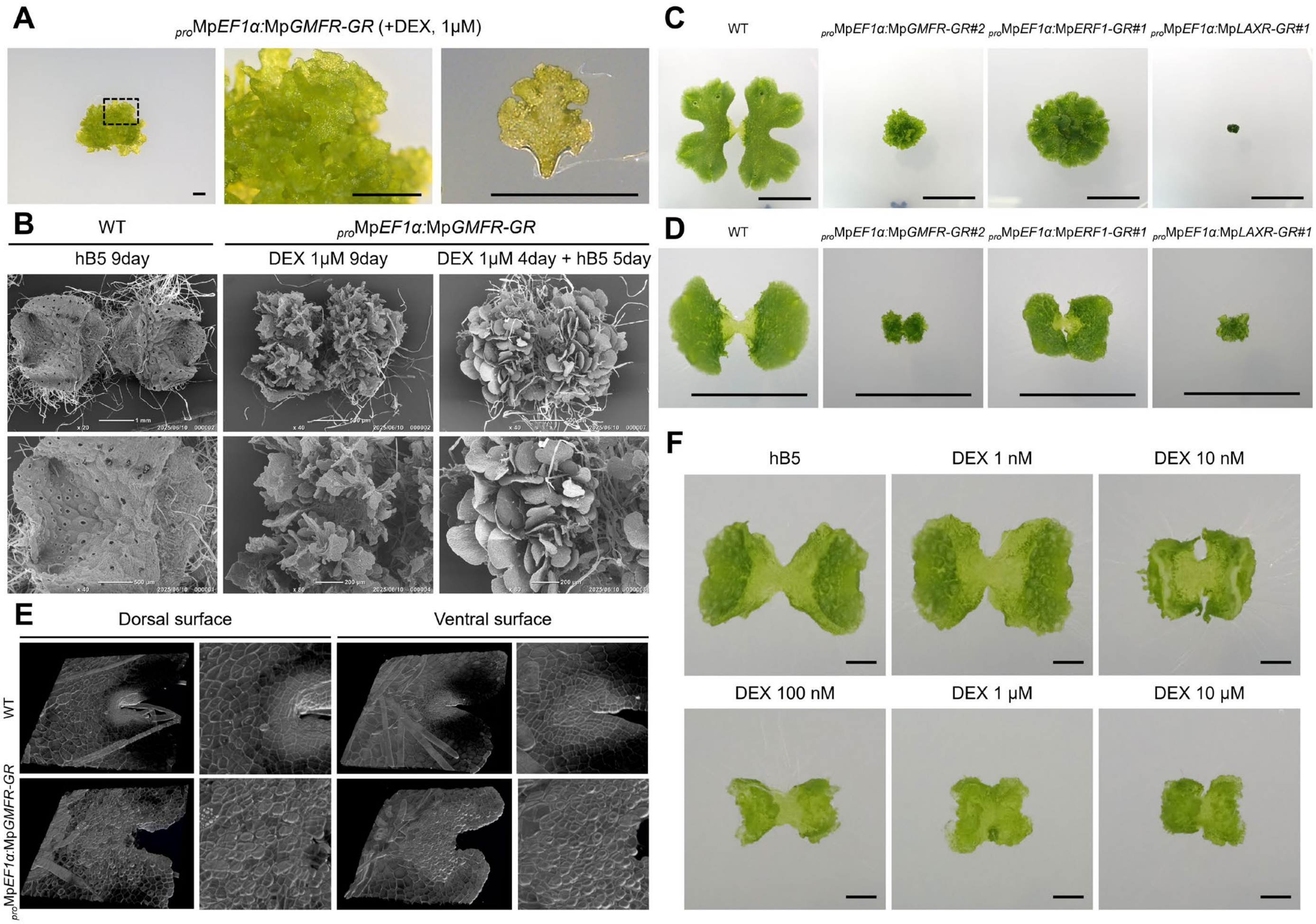

Figure S4. Overexpression phenotypes of Mp*GMFR* and its homologs. Related to Figures 3–5.
(**A**) Morphology of $_{pro}$Mp*EF1α:*Mp*GMFR-GR* plant grown on 1 μM DEX-containing medium for 9 days. The middle panel shows a close-up of the inset from the left panel. The right panel shows a magnified view of the tissue excised from the plant shown in the middle panel. Scale bars, 1 mm. (**B**) SEM images of the surface of 9-day-old thalli. Genotypes and growth conditions are indicated above the panels. Scale bar lengths are indicated in each panel. (**C** and **D**) Effects of inducible overexpression of Mp*GMFR*, Mp*ERF1* and Mp*LAXR*/Mp*ERF20*. Overall morphology of plants grown on 1 μM DEX-containing medium for 14 days (**C**) and those grown on 1 μM DEX-containing medium for 4 days followed by culture on DEX-free medium for 5 days (**D**). Scale bars, 1 cm. (**E**) Dorsal and ventral surfaces are shown from the 3D-reconstructed images of the apical notches in 3-day-old wild-type and $_{pro}$Mp*EF1α:*Mp*GMFR-GR* gemmalings. Right panels show magnified view of the areas indicated by dashed boxes in left panels. Cell walls were stained with SR2200. (**F**) Morphology of 7-day-old $_{pro}$Mp*EF1α:*Mp*GMFR-GR* plants grown from gemmae on media supplemented with indicated concentrations of DEX. Scale bars, 1 mm.

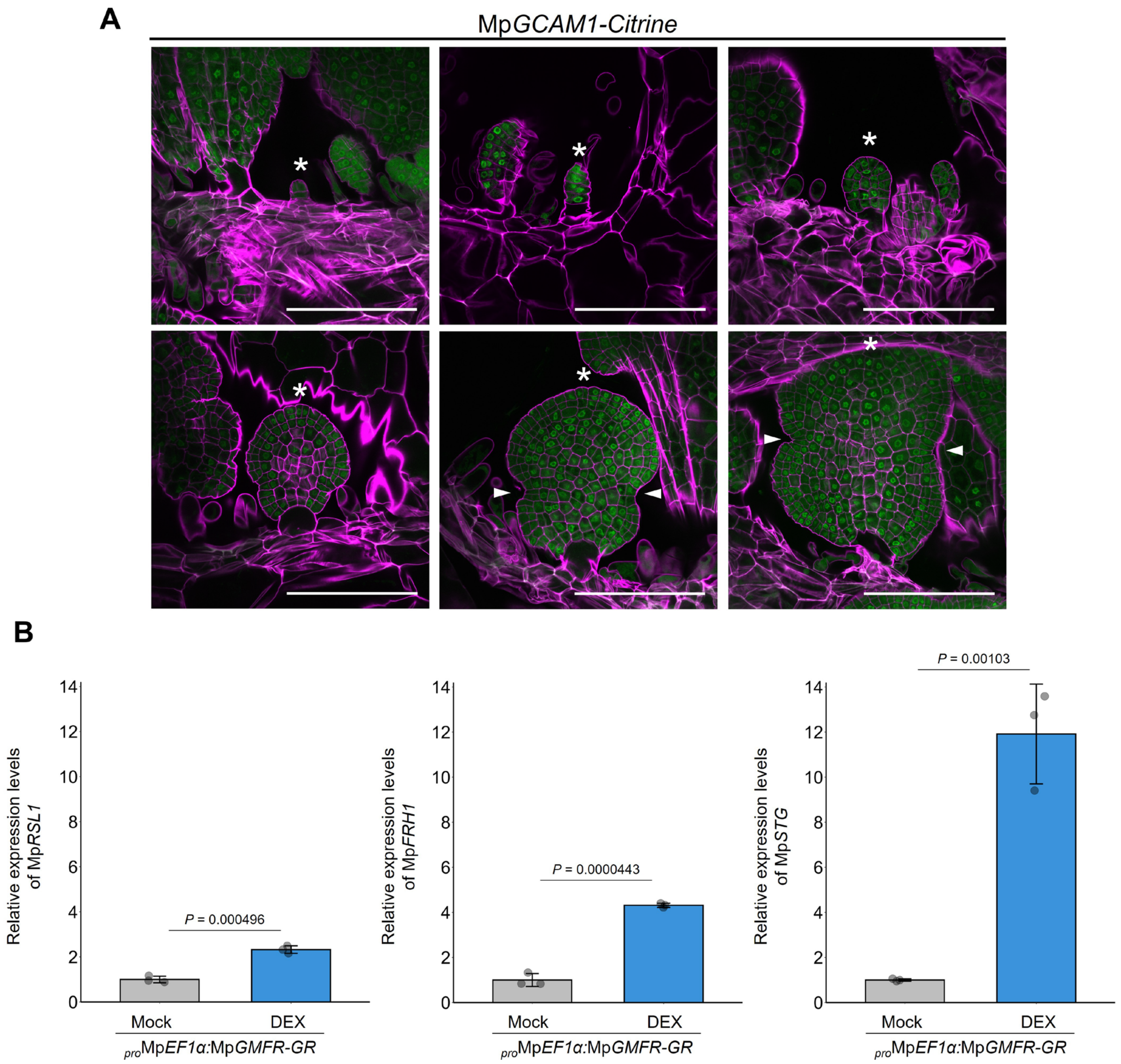

Figure S5. Expression analysis of known genes for gemma development. Related to Figures 2 and 6.
(**A**) Confocal imaging of Mp*GCAM1-Citrine* plants in developing gemmae in 14-day-old plants. Asterisks indicate developing gemmae. Arrowheads indicate apical notches. Cell walls were stained with SR2200. Scale bars, 100 μm. (**B**) Relative expression levels of Mp*RSL1,* Mp*FRH1* and Mp*STG* in $_{pro}$Mp*EF1α*:Mp*GMFR-GR* plants grown with mock or 1 μM DEX-containing medium for 4 days. Data are represented by mean and SD (bars) with individual data points (dots). Statistical significance was determined by Student's *t*-test. *P*-value is indicated above each pair of bars.

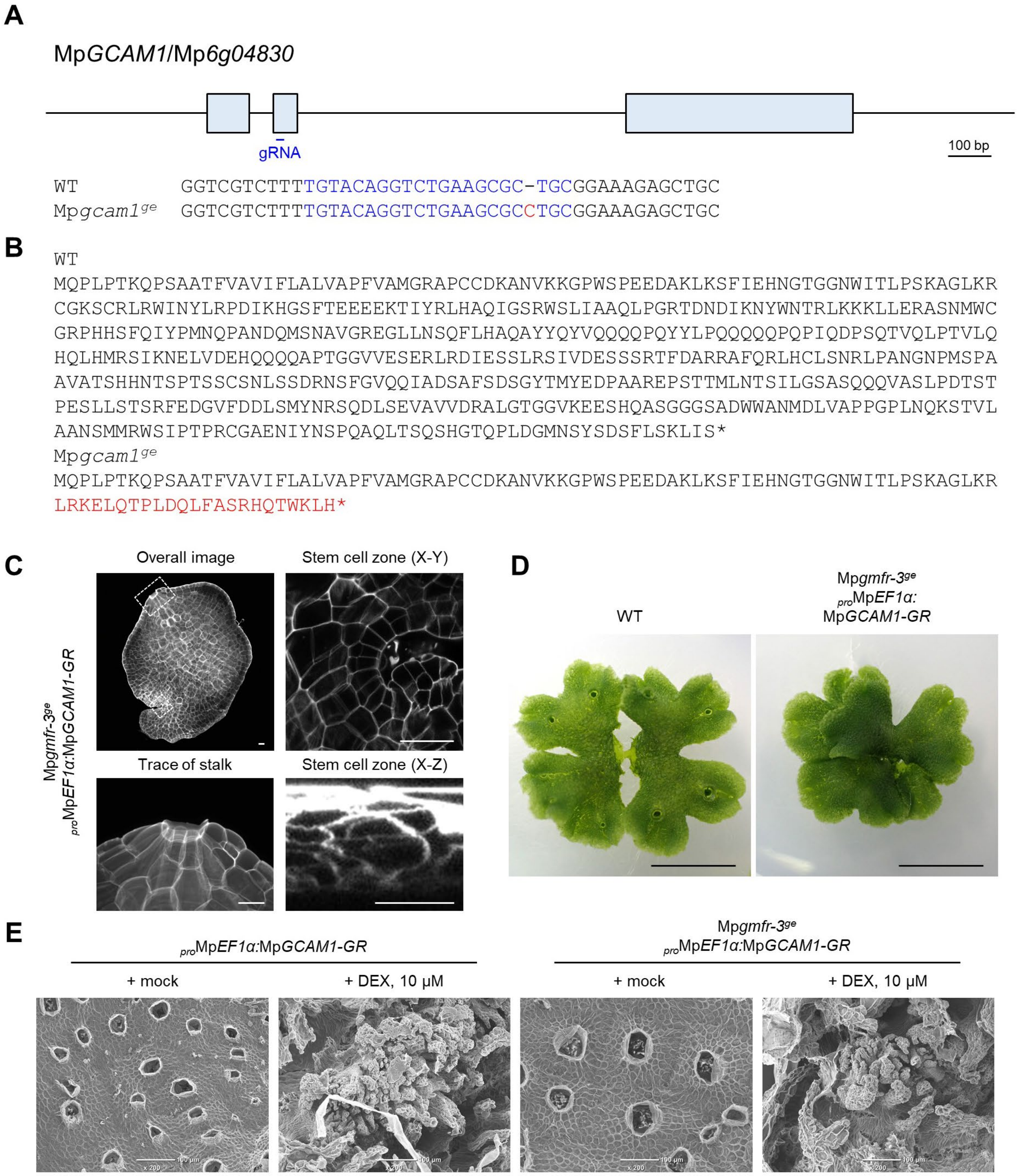

Figure S6. Functional analysis of Mp*GCAM1*. Related to Figure 6.
(**A**) Structure of Mp*GCAM1*/Mp6g04830 locus with the position of designed guide RNA (gRNA). Exons are shown as boxes. Genotyping of a genome editing allele is indicated below. gRNA sequence is in blue. An inserted base is in red. (**B**) Deduced MpGMFR protein sequences for WT and mutant. The sequence different from WT is in red. An asterisk indicates translational termination. (**C**) Confocal imaging of a gemma formed in Mp*gmfr-3*^{ge} $_{pro}$Mp*EF1α:*Mp*GCAM1-GR* plant by DEX treatment (top-left), containing a stem cell zone and a trace of stalk indicated in dashed boxes. The stem cell zone is shown in a single X-Y plane (top-right) and a reconstructed X-Z orthogonal view (bottom-right) derived from Z-stack images. The trace of stalk is shown in a 3D-reconstructed view (bottom-left). Scale bars, 25 µm. (**D**) Sixteen-day-old thalli grown from gemmae of WT and Mp*gmfr-3*^{ge} $_{pro}$Mp*EF1α:*Mp*GCAM1-GR*. Scale bars, 1 mm. (**E**) SEM images of the thallus surface of 14-day-old plants grown from explants including an apical notch on mock or 10 µM DEX-containing medium. Scale bars, 100 µm.

Primer Names	Sequence (5' to 3')	Purpose
**MpGMFRg_108F_A**	ctcgGCTGGCTCGACGAGCGTAGC	Genome editing
**MpGMFRg_108F_B**	aaacGCTACGCTCGTCGAGCCAGC	Genome editing
**MpGMFR_CDS_341R**	GACTTCAGATGGTCCTCCTCGAG	Genotyping
**MpGCAM1g_363f_A**	ctcgTGTACAGGTCTGAAGCGCTG	Genome editing
**MpGCAM1g_363f_B**	aaacCAGCGCTTCAGACCTGTACA	Genome editing
**MpGCAM1_CDS_617R**	GTAAACCCTACAAATGCGAGCAG	Genotyping
**MpGMFR_amiR_F**	cactTTTTCCCGTTTAATTGGGCCCACTGAGGAGCTCCTCAGAGACCTT GACAGGCTCCGTAGCGGGCCCGATTAAGCGGGGAAA	Artificial microRNA
**MpGMFR_amiR_R**	ctccTTTCCCCGCTTAATCGGGCCCGCTACGGAGCCTGTCAAGGTCTCT GAGGAGCTCCTCAGTGGGCCCAATTAAACGGGAAAA	Artificial microRNA
**MpGMFR_prom_F**	caccGCAGGGAGATTAATGGACAGGCG	pENTR cloning
**MpGMFR_prom_R**	GGATACTCCTATCGAAGAAACGACCG	pENTR cloning
**MpGMFR_CDS_F**	gcaggctccaccatgATGGCCTTGCATGATGTCCTC	pENTR cloning
**MpGMFR_CDS_R_+stop**	gtctagatatctcgaTCACAGACTGTAGGATCGCCATTG	pENTR cloning
**MpGMFR_CDS_R_-stop**	gtctagatatctcgaCAGACTGTAGGATCGCCATTGTTG	pENTR cloning
**MpGMFR_prom_F_InFusion_XbaI**	caagcttgggtctagaTTTAGCGACCGTGAAGAGGTGC	Insertion into Gateway Vector
**MpGMFR_prom_R_InFusion_XbaI**	tgttgataactctagaCTTCTAATCTTAACCGTGATCTTCTCTTCG	Insertion into Gateway Vector
**MpGMFR_CDS_gRNAres_F**	CTGGCTCGACGAGCGGAGTTGGAGATCTTGCGC	pENTR cloning
**MpGMFR_CDS_gRNAres_R**	GCGCAAGATCTCCAACTCCGCTCGTCGAGCCAG	pENTR cloning
**MpERF1_CDS_F**	gcaggctccaccatgATGGCTTTGAGGGACGAAGAGC	pENTR cloning
**MpERF1_CDS_R_-stop**	gtctagatatctcgaGGCCCGACTGCACAATGG	pENTR cloning
**MpLAXR_CDS_F**	gcaggctccaccatgATGGTGGGGAGGAAGCTGG	pENTR cloning
**MpLAXR_CDS_R_-stop**	gtctagatatctcgaCATGGGAACTAAAAGAAGAG	pENTR cloning
**MpGMFR_rt_F**	TGTATCGGCTGCCATGACTCC	RT-qPCR
**MpGMFR_rt_R**	AGCCGCAGACACTGGGATTG	RT-qPCR
**MpGCAM1_rt_F**	TGGATCAATTATTTGCGTCCCGAC	RT-qPCR
**MpGCAM1_rt_R**	ACCATCTGCTGCCAATTTGAGC	RT-qPCR
**MpRSL1_rt_F**	AGATGAGTCTGGGGCAACC	RT-qPCR
**MpRSL1_rt_R**	CGCTTTAGAGTGGTAGGCGC	RT-qPCR
**MpFRH1_rt_F**	GGGCTGCAGCACAAATCTAGATTC	RT-qPCR
**MpFRH1_rt_R**	GTTCACTGATGCACCGAACTTGG	RT-qPCR
**MpSTG_rt_F**	AACTCGTCCAAGATCACCATCC	RT-qPCR
**MpSTG_rt_R**	CAAGAGAGTAGATGCAGACGCT	RT-qPCR
**MpAPT_rt_F**	CGTCGTTGTTGGAATTGAAGC	RT-qPCR
**MpAPT_rt_R**	TTCTTGGGCTTTCGTTGAGG	RT-qPCR

Table S1. Primers. Related to STAR methods.
Small letters denote additional sequences for ligation, cloning and In-Fusion reaction.
